# Role modelling in professional identity formation: a systematic scoping review

**DOI:** 10.1186/s12909-023-04144-0

**Published:** 2023-03-29

**Authors:** Eugene Yong Hian Koh, Kai Kee Koh, Yaazhini Renganathan, Lalit Krishna

**Affiliations:** 1 Singapore Armed Forces, 303 Gombak Drive, Singapore, 669645 Singapore; 2 Yong Loo Lin School of Medicine, National University of Singapore, NUHS Tower Block, 1E Kent Ridge Road, Level 11, Singapore, 119228 Singapore; 3 Division of Supportive and Palliative Care, National Cancer Centre Singapore, 11 Hospital Dr, Singapore, 169610 Singapore; 4 Division of Cancer Education, National Cancer Centre Singapore, 11 Hospital Dr, Singapore, 169610 Singapore; 5 Lien Centre for Palliative Care, Duke-NUS Medical School, Singapore 8 College Road, Singapore, 169857 Singapore; 6 Palliative Care Institute Liverpool, Academic Palliative & End of Life Care Centre, University of Liverpool, Liverpool, UK; 7 Cancer Research Centre, University of Liverpool, 200 London Rd, Liverpool, L3 9TA UK; 8 Duke-NUS Medical School, National University of Singapore, College Rd, Singapore, 169857 Singapore; 9 Centre of Biomedical Ethics, National University of Singapore, 21 Lower Kent Ridge Rd, Singapore, 119077 Singapore; 10 The Palliative Care Centre for Excellence in Research and Education, PalC, PalC c/o Dover Park Hospice, 10 Jalan Tan Tock Seng, Singapore, 308436 Singapore

**Keywords:** Role model, Mentoring, Mentoring umbrella, Professional identity formation, Ring Theory of Personhood

## Abstract

**Background:**

Role modelling’s pivotal part in the nurturing of a physician’s professional identity remains poorly understood. To overcome these gaps, this review posits that as part of the mentoring spectrum, role modelling should be considered in tandem with mentoring, supervision, coaching, tutoring and advising. This provides a clinically relevant notion of role modelling whilst its effects upon a physician’s thinking, practice and conduct may be visualised using the Ring Theory of Personhood (RToP).

**Methods:**

A Systematic Evidence Based Approach guided systematic scoping review was conducted on articles published between 1 January 2000 to 31 December 2021 in the PubMed, Scopus, Cochrane, and ERIC databases. This review focused on the experiences of medical students and physicians in training (learners) given their similar exposure to training environments and practices.

**Results:**

12,201 articles were identified, 271 articles were evaluated, and 145 articles were included. Concurrent independent thematic and content analysis revealed five domains: existing theories, definitions, indications, characteristics, and the impact of role modelling upon the four rings of the RToP. This highlights dissonance between the introduced and regnant beliefs and spotlights the influence of the learner’s narratives, cognitive base, clinical insight, contextual considerations and belief system on their ability to detect, address and adapt to role modelling experiences.

**Conclusion:**

Role modelling’s ability to introduce and integrate beliefs, values and principles into a physician’s belief system underscores its effects upon professional identity formation. Yet, these effects depend on contextual, structural, cultural and organisational influences as well as tutor and learner characteristics and the nature of their learner-tutor relationship. The RToP allows appreciation of these variations on the efficacy of role modelling and may help direct personalised and longitudinal support for learners.

**Supplementary Information:**

The online version contains supplementary material available at 10.1186/s12909-023-04144-0.

## Introduction

Role modelling in medical education boosts cognitive skills [1], shapes moral values [2, 3], moulds professional practice [6–8], and instils professional, clinical, sociocultural expectations, standards of practice, professional codes of conduct, goals, roles and responsibilities [4, 5]. However, despite this burgeoning array of functions, it is role modelling’s ability to shape “what being a good doctor means and the manner in which he or she should behave” [4, 5] in “the foundational process one experiences during the transformation from lay person to physician” (professional identity formation or PIF) [5, 6] that has garnered the most attention.

However, till now, deeper evaluation of role modelling has been hampered by a lack of a clear definition [7], continued conflation with other practices, and focus upon the learner-tutor dyad, often to the exclusion of wider contextual considerations, learner and tutors related factors [8]. There has also been a failure to consider role modelling’s unintended, long-lasting negative and positive effects [7].

Krishna, Toh [8] and Radha Krishna, Renganathan [9]’s concepts of the mentoring umbrella offers a unique opportunity to study role modelling in a new light. The mentoring umbrella posits that role modelling is part of a spectrum of intertwined approaches including mentoring, supervision, coaching, tutoring and advising [10, 11]. As is increasingly reported, role modelling in the mentoring umbrella is often applied in tandem with one or more of these approaches. This perspective allows accounts of role modelling to be studied in tandem, negating the need to unpick one from the other for closer scrutiny. In light of this, a review into what is known of role modelling is proposed to better employ, structure, support and oversee its use in medical training.

### Theoretical framework

Role modelling’s ability to shape professional identity, thinking, feeling, attitudes and practice is best described by a constructivist ontological and relativist epistemological position. This lens also best captures its part in the guided immersion of medical students, the experiential learning of physicians in training, and the wider clinical, professional, environmental and sociocultural influences upon role modelling.

This theoretical lens also allows use of Radha Krishna and Alsuwaigh [12]’s concept of the Ring Theory of Personhood (RToP) to capture the wider effects of the mentoring umbrella [13] on professional, personal and research development and professional identity formation (PIF) [15–19]. Appreciation of these particularised effects upon the various aspects of a medical student or physician in training (henceforth learner) will allow better appreciation of the mechanism behind role modelling.

### The theoretical lens: the ring theory of personhood

**Fig. 1:**
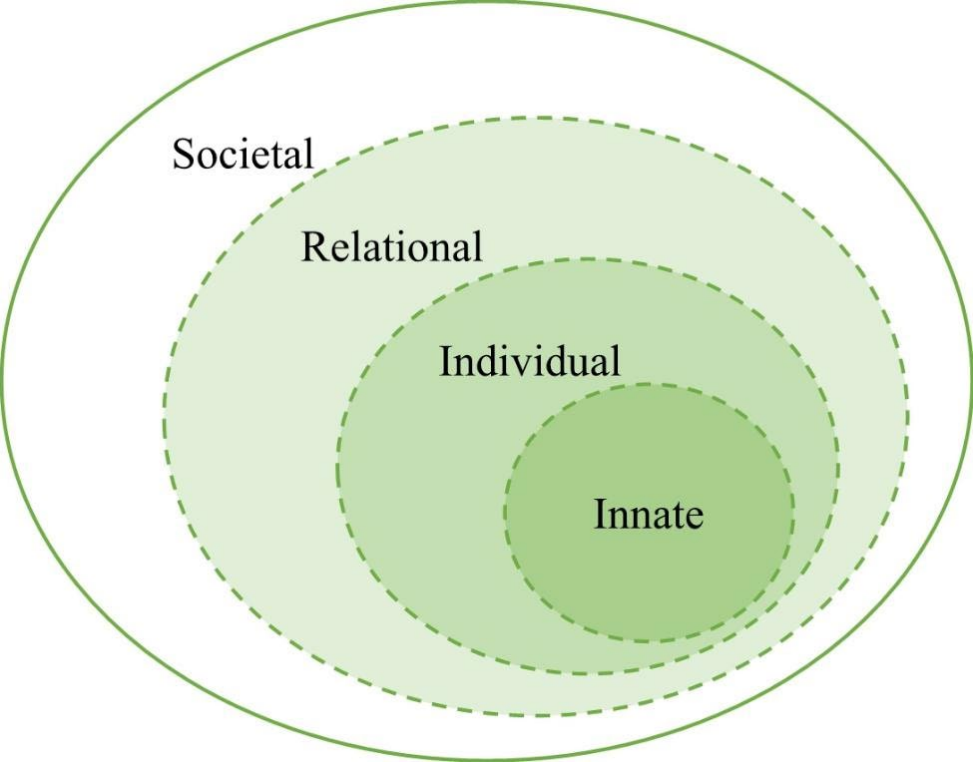
The Ring Theory of Personhood

Previous reviews [14, 15, 16, 17, 18] have revealed and mapped the inevitable tensions or ‘conflicts’ between the rings of the RToP in the face of moral distress and identity formation, and their impact upon self-concepts of personhood and identity. It is posited that the RToP will forward a better understanding of how role modelling inculcates new professional values, beliefs, and principles and shapes a learner’s professional identity.

The RToP suggests that personhood is composed of the Innate, Individual, Relational and Societal domains (Fig. 1). Each ring possesses a belief system containing the patient’s values, beliefs and principles. The Innate Identity considers religious, gender, cultural, community-based beliefs, moral values and ethical principles [19–23]. The Individual Identity encompasses personal values, beliefs, and personalities [24–27] whilst the Relational and Societal Identities pivot on familial and societal values, beliefs, expectations, and principles, respectively [24–27]. The integration of new experiences, insights, norms, codes of practice and ideals into current values, beliefs and principles that underpin a learner’s identity is likely to cause ‘conflict’ within the rings (disharmony) and between the rings (dyssynchrony) [28]. Understanding the tensions will enhance appreciation of the mechanism by which role modelling integrates and attends to ‘conflicts’ within and between the rings.

## Methodology

A Systematic Evidence Based Approach guided systematic scoping review (henceforth SSR in SEBA) [29–42] is used to study the effects of role modelling amongst medical students and physicians in training where increasing use of experiential learning and clinical integrated programs see them exposed to similar role modelling, training cultures, practices and environment, and hidden curricula. We recognise that role modelling will differ amongst specialists, consultants and attendings who are not involved in formal training programs and thus exclude them from this study.

**Fig. 2:**
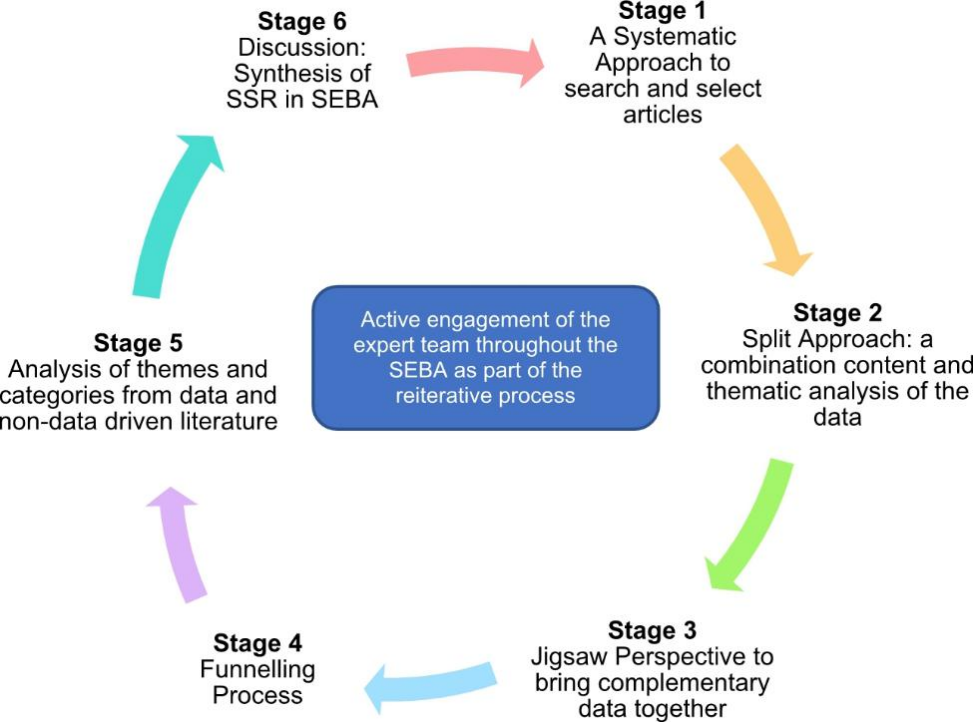
The SEBA Process

This SSR in SEBA is overseen by an expert team comprising medical librarians from the Yong Loo Lin School of Medicine (YLLSoM) and the National Cancer Centre Singapore (NCCS), and local educational experts and clinicians at NCCS, the Palliative Care Institute Liverpool, YLLSoM and Duke-NUS Medical School who guide, oversee and support all stages of SEBA to enhance reproducibility and accountability of the study process (Fig. 2).

### Stage 1 of SEBA: systematic approach

The research and expert teams set the overarching goals, study population, context and remediation programs to be evaluated and were guided by the Population, Intervention, Comparison, Outcome and Study Design (PICOS) elements of the inclusion criteria [43, 44] Table 1.

**Table 1:** PICOs, Inclusion Criteria and Exclusion Criteria Applied to Database Search

PICOS	Inclusion Criteria	Exclusion Criteria
**Population**	• Medical students, junior doctors and residents in all specialities and subspecialities of psychiatry, medicine, surgery, paediatrics, family medicine and obstetrics and gynaecology • In formal training programs or structured and assessed longitudinal programs, including residency and advanced training programs, specialist training, surgical training, and other speciality and subspeciality training programs.	• Allied health specialties such as dietetics, nursing, psychology, chiropractic, midwifery, social work • Non-medical specialties such as clinical and translational science, veterinary, dentistry • Not in training programs such as attendings, consultants and or physicians who have exited structured training programs.
**Intervention**	• Role modelling • Supervision • Coaching • Teaching • Tutoring • Novice mentoring involving junior physicians, residents and/or medical students mentored by senior clinicians aimed at advancing the professional and/or personal development of the mentee o Mentoring processes o Mentor factors o Mentee factors o Mentoring relationship o Host organization o Outcomes of mentoring o Barriers to mentoring o Mentoring structure o Mentoring framework o Mentoring culture o Mentoring environment	• Peer mentoring, Near-peer mentoring, mentoring for leadership, mentoring patients or mentoring by patients, interdisciplinary mentoring
**Comparison**	• Comparisons accounts of mentoring between mentoring programs, editorials and perspective, reflective, narratives and opinions pieces	
**Outcome**	• Personal outcomes of mentoring such as values, beliefs, identity as a medical professional etc. • Professional development outcomes such as on career choices (including academia positions/careers)	• Papers that did not discuss impact of role modelling on personal or professional development outcomes
**Study design**	• All study designs are included o Descriptive papers o Qualitative, quantitative, and mixed study methods o Systematic review, literature reviews, and narrative reviews • Perspectives, opinion, commentary pieces, and editorials • Year: 1st Jan 2000–31st December 2021	

Given that this review sees role modelling as part of the mentoring umbrella and intimately entwined with practices such as mentoring, supervision, coaching, tutoring, and teaching, these terms were included in the general search. However, given resource limitations and data from our recent reviews of the various constituents of the mentoring umbrella separate searches of each of these practices were not conducted. Focus was upon role modelling and any accounts of its combined use with other aspects of the mentoring umbrella.

Independent searches were carried out between 18th October 2021 and 17th January 2022. The searches involved PubMed, Scopus, Cochrane, ERIC and grey literature databases (GreyLit, OpenGrey, and Web of Science). Additional ‘snowballing’ of references of the included articles ensured a more comprehensive review of the articles [45] This search was carried out between 17th January 2022 and 24th April 2022.

Using an abstract screening tool, the research team independently reviewed abstracts to be included and employed ‘negotiated consensual validation’ to achieve consensus on the final list of articles to be included [46].

### Stage 2 of SEBA: split approach

The Split Approach [17, 47] sees concurrent thematic and directed content analysis of the included full-text articles by three independent teams. The first team summarised and tabulated the included full-text articles (Appendix A).

#### Thematic analysis and directed content analysis

Using Braun and Clarke [48]’s approach to thematic analysis, the second team ‘actively’ read the included articles to find meaning and patterns in the data and achieved consensus on the final list of themes. [49–53].

Using Hsieh and Shannon [54]’s approach to directed content analysis, the third team identified categories from Cruess and Cruess [55]’s article, “The Development of Professional Identity” and achieved consensus on the final list of categories.

The final codes were compared and discussed with the final author who checked the primary data sources to ensure that the codes made sense and were consistently employed. Any differences in coding were resolved.

‘Negotiated consensual validation’ was used as a means of peer debrief in all three teams to further enhance the validity of the findings [56].

### Stage 3 of SEBA: jigsaw perspective

The Jigsaw Perspective employs Phases 4 to 6 of France et al. France, Wells [57]’s adaptation of Noblit et al. Noblit and Hare [58]’s seven phases of meta- ethnographic approach to view the themes and categories as pieces of a jigsaw puzzle where overlapping/complementary pieces are combined to create a bigger piece of the puzzle referred to as themes/categories. This process would see themes and subthemes compared with the categories and subcategories identified. These similarities were verified by comparing the codes contained within them. If they are complementary in nature, the subtheme and subcategory are combined to create a bigger piece of the jigsaw puzzle.

### Stage 4 of SEBA: funnelling

Themes/categories were compared with the tabulated summaries [57, 58]. These funnelled domains created from this process formed the basis of the discussion’s ‘line of argument’.

## Results

**Fig. 3:**
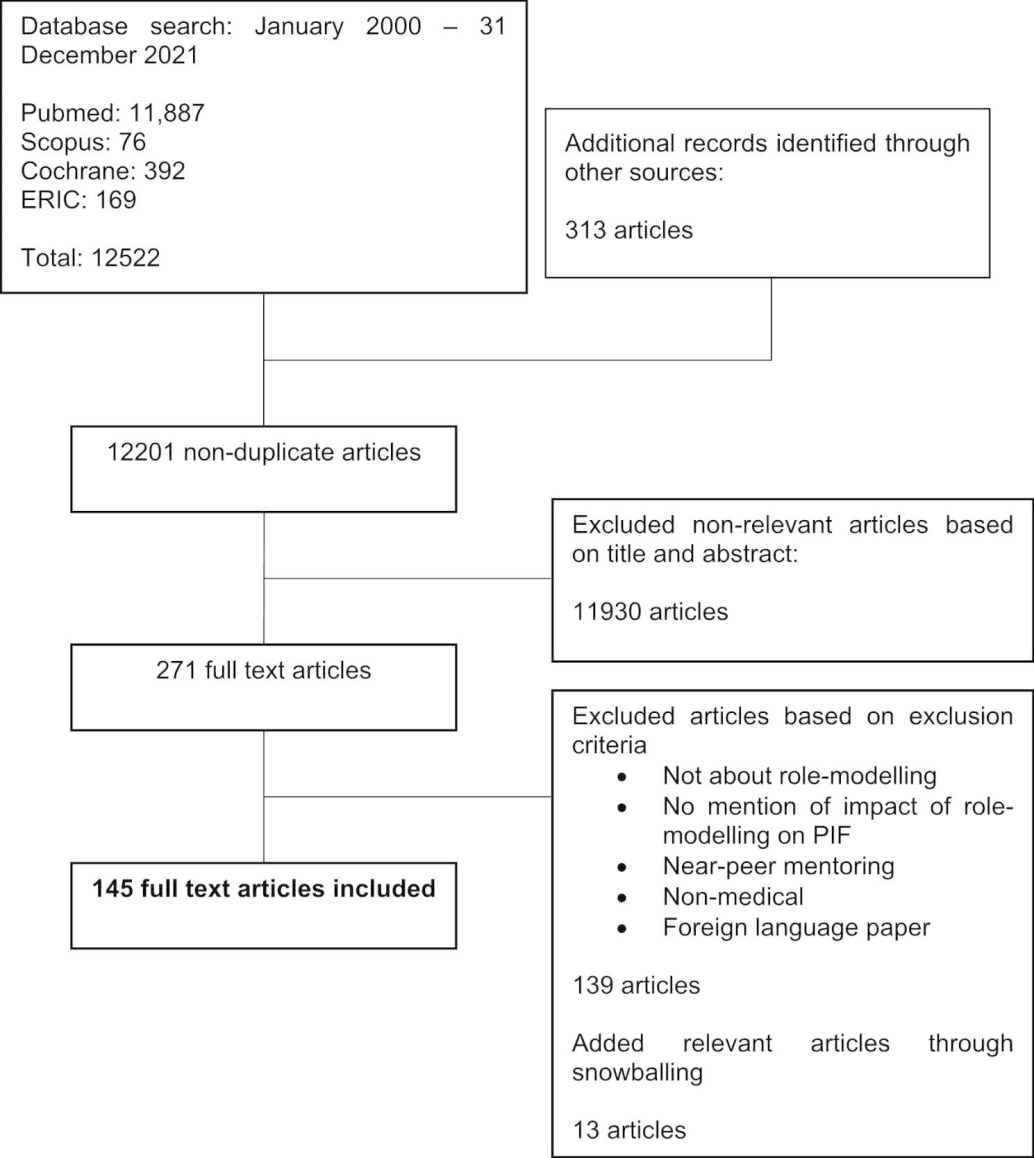
PRISMA Flowchart

A total of 145 articles were included (Fig. 3). Sixty eight articles explored role modelling in the undergraduate context, 36 were in the postgraduate context, and 41 explored role modelling in both undergraduate and postgraduate settings.

The themes identified were theories, indications, characteristics, impact and influences on the role modelling process. The categories identified were theories, characteristics, indications, influences and impact of role modelling.

**Fig. 4:**
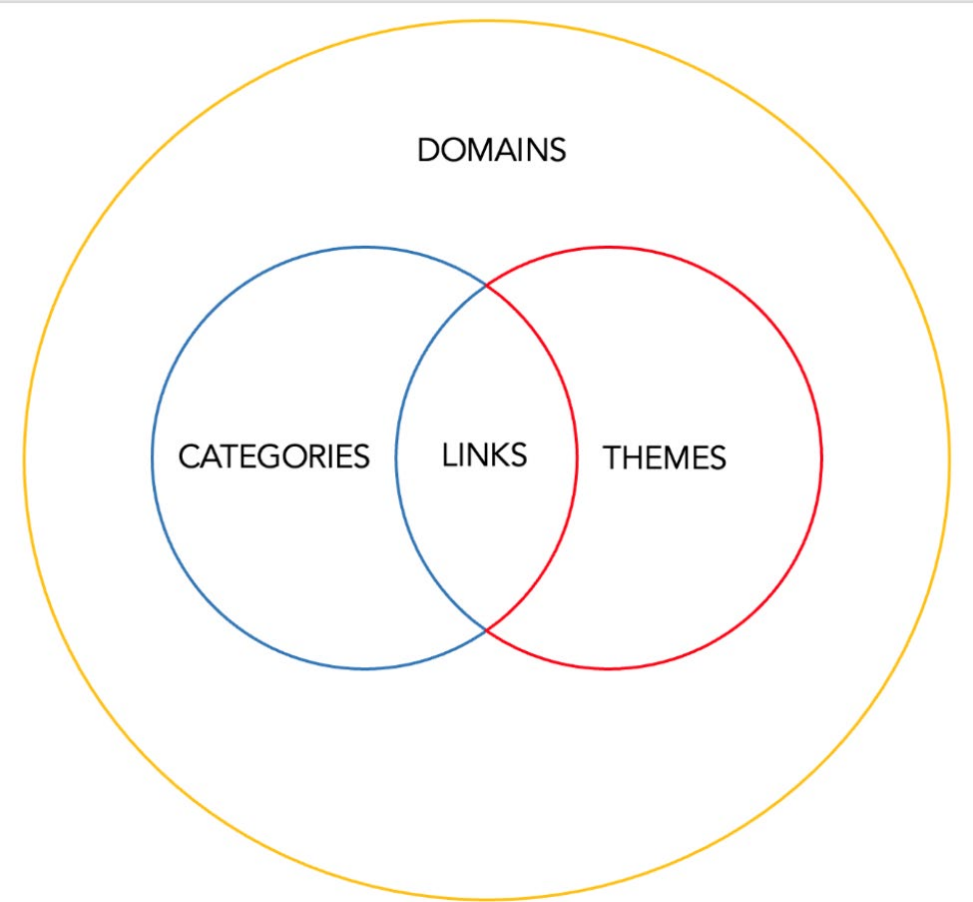
Domains, Categories, Themes

The funnelled domains (Fig. 4) created from the combination of the themes and categories were (1) existing theories, (2) definitions, (3) indications, (4) characteristics of role models, (5) the impact of role modelling.

### Domain 1. existing theories

Most current theories are built on the notion that role modelling hinges on active observation of the role model’s personal, clinical, and/or social circumstances, practice, attitudes, decisions and skills; reflection on these observations; translation of these insights into principles and actions; and integration of these insights into practice, thinking, attitudes, skills, deliberations and conduct [59–76]. Many of these theories focus on learner attitudes, belief system, narratives, clinical experiences, contextual considerations and positive outcomes of role modelling [1, 60, 61, 63, 64, 66, 67, 69, 77–118]. There is little consideration for the wider sociocultural, programmatic and practical factors impacting role modelling.

### Domain 2. definitions

Krishna et al. [7]’s review describe role modelling as “a process which may be formal or informal [1, 59, 63–70, 80, 81, 84–90, 92, 94, 97, 100–105, 108, 116, 119–146], immediate or delayed (requiring post-reflection) [59, 60, 65, 67, 69, 81, 86, 88, 89, 94, 98, 101, 106–108, 110, 114, 121, 142, 146–149], involving seniors, peers, or others within the profession as a role model [1–3, 59, 61, 62, 65, 67, 68, 70–72, 74, 76, 89, 91, 94, 97, 100, 102–106, 108, 109, 113, 114, 116, 118, 132, 134, 135, 137, 138, 141–146, 148–168], advertent or inadvertent by both the role model or the learner [1, 59, 60, 64, 66, 68–70, 92, 102, 103, 106–108, 110, 112–114, 116, 122, 134, 141, 142, 147, 150], and clinical or non-clinical [1–3, 59, 61, 62, 64, 66, 67, 70–76, 82, 83, 85, 89–91, 97, 99, 100, 102–106, 108, 109, 111–114, 116, 118, 120, 126, 131, 137, 141–146, 148–150, 152, 154, 159–174], in which positive or negative behaviours, actions or attitudes [1–3, 59–64, 66, 67, 69–72, 74, 77–118, 141–146, 148, 149, 159–168] are emulated or rejected by the learner” [1, 60, 61, 67, 69–71, 91, 100, 106–108, 110–112, 114, 115, 117, 141, 142, 150, 162, 167, 175].

### Domain 3. indications

Passi et al. [111]’s BEME Guide on role modelling, describes three key indications for role modelling.- transmitting professional behaviours [59–62, 65, 66, 68, 76, 77, 82, 100–103, 106, 108, 111, 114, 115, 121, 130, 135, 140, 143, 146, 148, 152, 161, 168], influencing the development of professional attributes of learners [1, 2, 59, 63, 64, 68–70, 72, 73, 75–77, 79, 81, 95–98, 100, 102, 103, 105, 108, 110–113, 115, 117, 119–122, 125, 126, 128, 132, 134, 135, 141, 144, 147, 148, 150, 152–154, 156, 161, 171, 172, 176–178] and influencing career aspirations of learners [59, 60, 70, 71, 73, 75–78, 80, 86, 88–90, 95, 98, 99, 102, 109, 111–113, 118, 126, 134, 135, 137, 138, 140, 143, 153, 155, 157, 158, 160, 162, 166, 169, 170, 174, 177, 179–185]. However, despite suggestions of a role in PIF, this has not been captured here.

### Domain 4. characteristics of role models

Effective role modelling depends on characteristics of the role models. Factors that draw learners to a role model include their personal characteristics, relatability, ability to build relationships with the learner, and their clinical and teaching competencies (Table 2).

**Table 2:** Characteristics of role models

*Positive*	*Negative*
**Clinical competencies**	
• Clinical knowledge and skills (107, 112, 118) • ‘diagnostic and clinical skills’ (111) • ‘comprehensive approach to management, treatment and investigations’ (111) • Communication with patients and staff (107, 174) • Sound clinical reasoning and decision making (107, 118)	• Insufficient medical knowledge (118) • Insensitive to the needs of patients (102) • Inadequate relations with patients (118) • Uncooperative interaction with health care workers (102, 118) • Focused on tutor-centred patient interactions in order to save time (102) • Inappropriate medical reasoning (118)
**Teaching skills**	
• Concern for student well-being (75) • Approachability (75, 77) • Inspirational (174) • Student-focused (174) • Knowledgeable (174) • Patience (174) • Aware and prepared for their roles as role models (59, 63–66, 73, 81, 85, 88–91, 93, 95, 96, 98, 99, 101, 103, 107, 110, 117, 119, 126, 130, 132, 134, 135, 137, 153, 154, 176, 177, 186, 187) • Keeping the teaching simple, clear, informative, well-organised, and well-illustrated (172) • Demonstrating professionalism in daily work, • Explicitly explaining to learners the rationale behind actions, • Guiding the reflective process of learners, and providing timely • And meaningful formative feedback (86, 88, 96, 98, 110, 119)	• Rarely give feedback (116) • Humiliation of students (102) • Demoralising to learners (109)
**Personal characteristics**	
• Empathy (77, 112, 150) • Respect (77, 112) • Effective interpersonal skills (107, 112, 150) • Compassion (77, 107) • Positive outlook (112) • Leadership (112) • Dedication (112) • Commitment to excellence (107, 112) • Altruism (150) • Honesty (107, 112) • Politeness (112) • Inspiring (112) • Enthusiasm (112) • Integrity (77, 107, 112) • Ethical and moral practice (77) • Care and compassion (77) • Punctuality, professionalism (77) • Commitment to job (77) • Honest communication (77) • Good listening (77) • Discipline (77) • Rule-following (77) • Charismatic individual (1, 60, 150) • Humanistic and collaborative relations with patients and colleagues (118) • External manifestations of professional (118) • Cooperation rather than competitiveness (109) • Gender or sexual identity (71, 72, 75, 159), race (72, 75), and personality (111) • Learners emulate role models that they feel are closer to their own present identity (71, 72, 74, 75, 111, 159)	• Poor interpersonal relations (1) • Lack of self-confidence (118) • Absence of leadership (118) • May be rude to patients, students or staff, and may exhibit condescending behaviour (77) • Lack of integrity (1) • Lack professionalism (77) • Inadequate external appearance (118)
**Institutional factors**	
• Promotes balanced working practices, • Incentivises tutors, • Provides them with ‘protected time’ to teach and role model (84, 94, 107, 121, 127, 134, 136, 153, 157) • Time for reflection (59, 60, 65, 67, 69, 81, 86, 88, 89, 94, 98, 101, 106, 107, 110, 114, 121, 146, 147) • Aligned implicit curriculum, or “hidden” or “informal” curriculum, with the explicit, or “formal” curriculum boosts positive role modelling(108), • Consistent approach (175) • Making behaviours more intentional (86, 88, 96, 98, 110, 119)	• Time pressures • Lack of protected time, • External stress, • Bureaucracy, • Conflicts between explicit and implicit curriculums (63, 79, 86, 88, 96, 98, 108, 110, 119, 123, 154, 175, 188)

### Domain 5. impact of role modelling through the lens of the RToP

Role modelling has an array of effects upon the learner. These effects are summarised in Table 3 for ease of review. Perhaps more significantly, the impact of these effects vary from learner to learner. The RToP explains that these differences are a result of ‘resonance’ between regnant belief systems and the practices, guidance, expectations, roles, responsibilities and standards being introduced [67, 100, 155] or ‘conflict’ which take the form of disharmony [84, 99–101] and/or dyssynchrony [84].

**Table 3:** The Impact of Role modelling

Innate	• Reassurance of success regardless of gender or gender identity (71, 159, 160, 181) • Tolerance (60, 69, 74, 80, 165) • Humanistic attitude (67, 87, 98, 100, 111, 116–118, 122, 126, 137, 141, 147, 152, 154, 169, 171, 189)
**Individual**	• Personal care and wellness (68, 75, 76, 100, 108, 128, 163, 178, 190) • Influences career choice (59, 60, 70, 71, 73, 75–78, 80, 86, 88–90, 95, 98, 99, 102, 109, 111–113, 118, 126, 134, 135, 137, 138, 140, 143, 153, 155, 157, 158, 160, 162, 166, 169, 170, 174, 177, 179–185) • Ability to build rapport and communicate with patients (68, 80, 85, 90–92, 96, 98, 101, 113, 117, 120, 126, 141, 145, 147, 152, 154, 178) • Communication with peers and colleagues (1, 68, 92, 102, 105, 109, 112, 118, 119, 126, 141, 144, 149, 150, 162, 165, 191, 192) • Communicating with juniors and students (68, 73, 97, 102, 112, 118, 120, 122, 126, 152, 162–165) • Career success (97, 160, 173) • Knowledge (66, 77, 82, 83, 85, 94, 96, 102, 113, 116, 120, 122, 137, 141, 154–156, 165, 169, 173, 174, 191, 193) • Teaching skills (73, 75–77, 80, 82, 85, 88–91, 96, 97, 100, 102, 104, 112, 122, 126, 131, 137, 151, 152, 154, 156, 157, 165, 170, 172, 174, 192–194) • Career satisfaction (71, 73, 76, 154, 155, 177) • Motivating and inspiring, positivity (66, 71, 73, 76, 80, 89, 90, 97, 102, 109, 111, 113, 116, 128, 153–155, 157, 165, 171, 189, 195) • Readiness to express feelings (1, 84, 116) • Humility (80, 91, 102, 108, 120, 137, 147, 150, 154, 171, 189) • Empathy (61, 67, 68, 77, 80, 85, 89, 91, 92, 96, 102, 108, 111, 113, 117, 122, 128, 130, 131, 144, 150, 154, 161, 176, 189, 190, 192) • Honesty/Integrity (1, 66, 68, 73, 80, 85, 90, 92, 97, 102, 103, 108, 113, 119, 128, 131, 137, 144, 147, 152–154, 157, 161, 162, 165, 170, 178, 189, 190, 193) • Respectfulness (68, 73, 77, 80, 82, 85, 92, 102, 108, 111–113, 117, 122, 128, 131, 142, 149, 151, 152, 154, 161, 162, 165, 178, 190, 192, 193) • Compassion (66, 67, 73, 76, 77, 80, 84, 85, 90–92, 97, 102, 108, 111, 113, 117, 122, 128, 131, 142, 144, 149, 152, 154, 161–163, 171, 174, 178, 189, 190, 192, 193) • Curiosity (92, 108, 178, 189) • Contending with Cynicism (60, 76, 92, 112, 115, 116, 125, 142, 144, 154, 164) • Dedication (66, 80, 102, 113, 137, 142, 161, 170, 189) • Self-improvement (68, 73, 85, 113, 152–154, 161) • Leadership (70, 71, 73, 80, 89–91, 102, 113, 118, 131, 153, 154, 157, 163, 189) • Commitment to excellence (73, 102, 108, 113, 153, 154, 157, 189) • Patience/calmness (85, 112, 122, 147, 154, 174, 192) • Altruism (111, 128, 154, 171, 190) • Responsibility (80, 119, 154, 190) • Resilience (128, 140, 154, 189) • Self-confidence (154)
**Relational**	• Relationship with family (71, 73, 128) • Able to balance work with familial duties and obligations (71, 73, 75)
**Societal**	• Maintaining a hierarchy in the profession (96, 109, 112, 116, 150, 165) • Treatment of juniors (66, 102, 104, 113, 130, 149, 150, 165, 172, 190) • Support of students (116, 163, 165) • Contribution to research (85, 89, 90, 118, 154, 162, 170, 173, 191) • Relationship with patients (61, 66, 76, 77, 80, 88, 96, 97, 100, 102, 111, 113, 116–118, 130, 137, 141, 142, 144, 145, 150, 153, 154, 162, 164, 165, 176) • Commitment to teach (66, 68, 80, 97, 102, 104, 116, 120, 122, 154, 157, 162, 163, 165, 192, 195) • Attitude towards collaboration, competition, cooperation, and collegiality (63, 64, 68, 85, 98, 108, 116, 141, 154, 169, 192) • Relationship with colleagues including allied health (1, 76, 77, 89, 90, 96, 97, 102, 105, 109, 111, 112, 118, 141, 144, 149, 150, 154, 162, 165, 192) • Relationship with students/juniors (66, 73, 82, 83, 88–91, 96, 111, 120, 122, 151, 153, 154, 157, 170) • Clinical competency (61, 64, 66, 67, 73, 75, 76, 82, 83, 85, 89, 90, 97, 99, 100, 102, 111–113, 116, 118, 120, 126, 131, 137, 141, 149, 150, 152, 154, 163, 167, 169–174) • Motivation to develop professionalism (1, 113, 116, 162) • Maintaining of patient confidentiality (68, 145) • Avoiding use of derogatory humour (112, 115, 125, 153, 196, 197) • Patient-centred care (67, 68, 75, 100, 137, 154, 171) • Not discriminating against certain patient profiles (139, 196, 197) • Proper disclosure of medical information or errors (66, 68, 92, 93) • Organisation, management, efficiency (64, 80, 87, 118, 128, 154, 170) • Not engaging in sexual harassment (66, 109) • Cost conscious care (127, 136)

### Stage 5 of SEBA: analysis of evidence and non-evidence driven literature

To address concerns about data from grey literature, which was neither quality-assessed nor necessarily evidence-based, the study team thematically analysed data from grey literature and non-evidence-based pieces such as letters, opinion and perspective pieces, commentaries and editorials drawn from the bibliographic databases separately and compared these themes against themes drawn from peer-reviewed, evidence-based data. Similar themes were reveal suggesting that non-evidence-based articles did not bias the analysis.

## Discussion

In answering its research question, this SSR in SEBA on role modelling amongst medical students and physicians in training reveals a wider concept of role modelling than previously theorised. Rather than hinging almost exclusively on the learner’s active observation, cognitive base, clinical insight, reflective practice and ability to integrate their new insights and reflections, this SSR in SEBA reveals tutor-dependent and context-specific considerations.

### Learner-centric considerations

The data suggests that there is more to learner-centric considerations than previously proposed. These include the learner’s narrative which informs the learner’s ‘internal decision-making processes’. These ‘internal decision-making processes’ include the ability to detect a learning moment (sensitivity); determine if what they are observing is of interest or relevance, a positive or negative experience or observation and if they should ponder on these experiences (judgement); whether they have the ability, time, competency, and motivation to integrate the lessons learnt into practice and address any dissonance that may arise (willingness); and whether they can and are able to balance other considerations at hand as these lessons are integrated (balance).

These ‘internal decision-making processes’ are influenced by several other factors. Like the learner’s narrative and belief systems, their clinical insights and cognitive base influence their attention, motivations, sensitivity, judgement, willingness, reflections, balance, and beliefs. The learner’s contextual considerations include their interpretation of regnant practice, social, cultural, familial, relational, existential and clinical factors, together with their belief systems which contain their personal values, beliefs and principles. These help to fashion their personal moral and ethical compass and their attitudes towards practices, conduct, skills and/or attitudes being role-modelled. This combination helps to mould their efforts as they attend to resonances, disharmony and/or dyssynchrony with their prevailing identity. Having the learner primed to receiving and valuing lessons learnt through purposeful role modelling also underlines the role of tutor-dependent and contextual factors.

### Tutor dependent factors

Tutor dependent factors include the tutor’s training, role modelling, feedback and support skills; their motivations, accessibility, experience, availability, ability; and their willingness to provide personalised, appropriate, timely and longitudinal support and feedback helps to configure the role-modelling process. At the heart of these considerations is the ability of the tutor to attract attention and change thinking. This is helped in part by the tutor’s position of influence, seniority, respect and ability to inspire the learner.

Tutor-dependent facets of role modelling also reveal setting-specific and tutor-learner relationship-contingent elements. Perhaps exemplifying this is the nature, significance, and depth of the pre-existing tutor-learner relationship and the presence of trust.

Overall tutor-dependent features reiterate the importance of tutor training, longitudinal tutor support, and the importance of preparing learners for their role modelling experiences.

### Contextual considerations

Contextual considerations pivot on structured and personalised aspects. A personalised approach ensures that the physician’s cognitive base, narratives, belief system, emotional state, clinical insights, and internal decision making processes are well-accomodated. It also ensures that mentored guidance in the observation, reflection, feedback and integration phases of role modelling is provided. This personalised aspect facilitates individualised guidance to help learners resolve conflicts between their current position and the values, beliefs and principles drawn from the role model. It also considers the learner’s social, cultural, familial, relational, existential, psycho-emotional state; and regnant environmental, sociocultural, professional, academic, clinical, research and curricular factors.

The structured aspect includes a consistent approach framed by the presence of clearly stipulated goals, clear expectations, standards of practice, codes of conduct, timelines, the presence of protected time for reflection and training, roles and responsibilities, and a training framework which includes ‘protected time’ for learning, reflection, debriefing, feedback and personalised and timely support. The structured aspect also considers the tutor’s accessibility, availability and motivations as well as their contextual and motivations. Overall, the structured approach introduces the importance of the formal program, the role of the host organisation and the structure of the program.

Cruess and colleagues (55, 65, 107, 198) underscore the significance of contextual considerations in role modelling by highlighting the socialisation process. The authors report that planned and structured role modelling carried out by trained tutors and overseen by a formal training program within a nurturing learning environment promotes integration of new values, practices, principles, beliefs, conduct and competencies [55, 65, 107, 198]. Perhaps less apparent but nonetheless critical is the role of the host organisation in ensuring effective balance between the personalisation and consistency (flexibility) of the role modelling process. In addition, the host organisation plays a pivotal role in ensuring oversight of support, feedback and remediation.

### Role modelling and professional identity formation

**Fig. 5:**
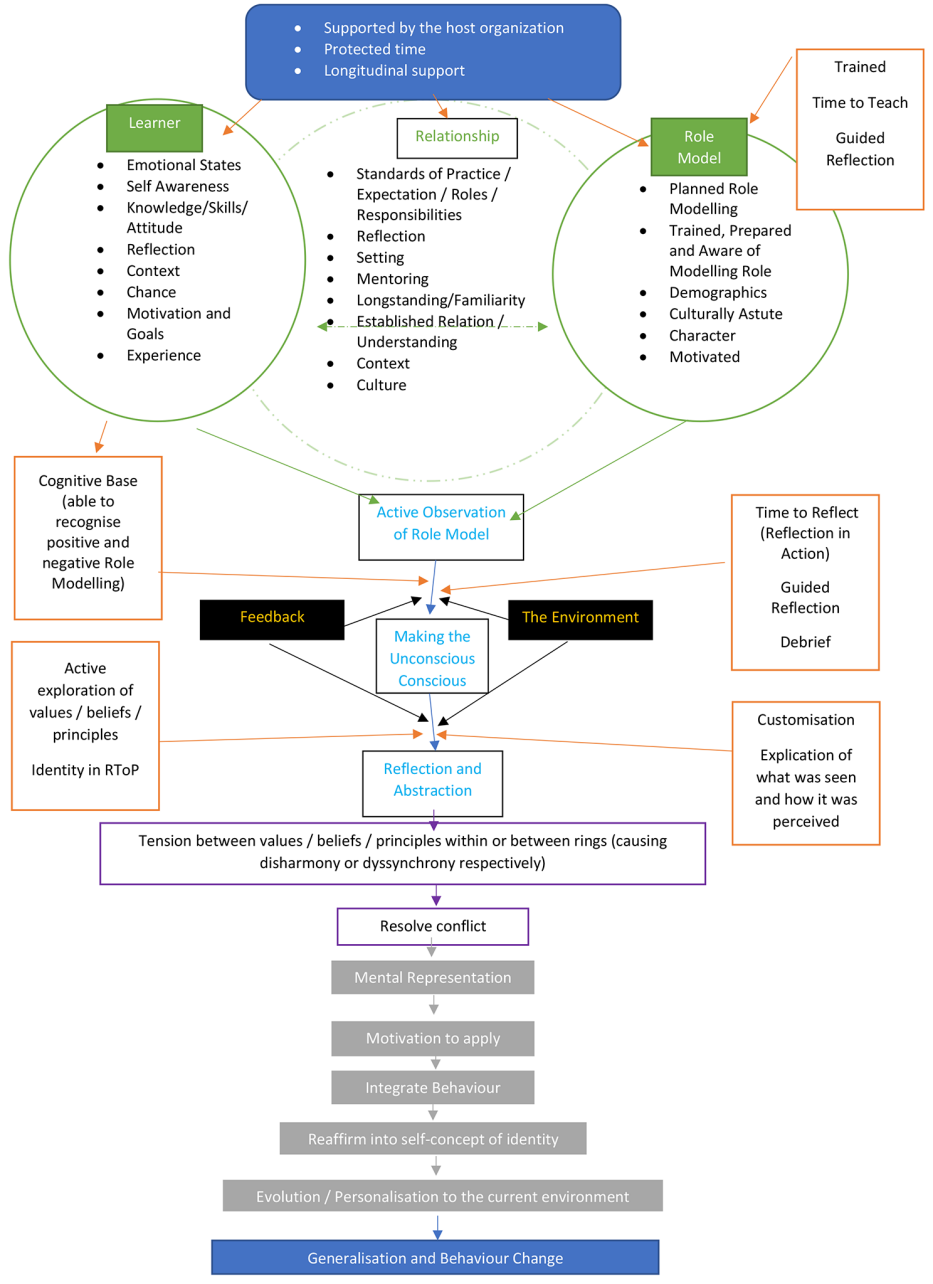
Mechanism of Role Modelling

Acknowledging that role modelling introduces new practices, skills, knowledge, attitudes and competencies that will guide how a learner will feel, think and act as a physician, role modelling clearly has a key role in nurturing the professional identity of learners. To achieve this, role modelling shapes their belief system which in turn influences how they see themselves. Built upon data accrued in this study, the mechanism behind role modelling is summarised in Fig. 5.

To attend to the effects of ‘disharmony’ and or ‘dyssynchrony’, adaptations are made to one’s belief system and professional identity. Learners must once again be motivated, willing and capable of balancing the wider considerations to achieve a viable professional identity. These changes in values, beliefs and principles and subsequent self-concepts of personal identities highlight the links between personal identity and PIF. This process also sees the learner’s interpretations and personalisation of what has been role modelled and how they have been employed.

## Limitations

There are several areas that limited this study. As the included articles comprised of reviews and primary studies, there were overlaps in the primary studies addressed in the reviews and those identified independently by the study team. Although the overlaps were considered in the analysis, no structured approach was undertaken.

Whilst there is a relative dearth of data on role modelling, perceiving role modelling as part of the mentoring umbrella has allowed it to be studied more widely together with similar practices. This allows a practical and modern perspective of current practices surrounding role modelling.

To ensure that this search approach is reproducible the SEBA approach was adopted. Whilst well evidenced in medical education and palliative care research, the need for three independent teams has restricted the focus of this study to medical students and physicians in training. Yet such a move may be justified by the fact that it builds on earlier studies on the mentoring umbrella, prevents conflation of data across different groups of healthcare professionals with distinct training practices and recognises the need to recognise the contextual influences of role modelling.

Focus upon reports published in English may have also restricted the search results. Similarly, focus upon role modelling published in the English language saw most of the data drawn from North America and the European countries that may not necessarily be transferable beyond these regions. However, given that practices in much of Asia are influenced by Western style education practices, it is entirely likely that the lessons learnt will be transferable albeit with some context specific adaptations to take into account local healthcare and education systems and sociocultural considerations.

## Conclusion

Role-modelling impacts PIF and influences personal identity, yet its true impact still needs further elucidation in order for it to be effectively guided and assessed. Missing also is evidence of the balancing process and adjustments to the belief system. Whilst it might be notions of identity patching and identity splinting such as those submitted by Pratt et al. [199] at play, it is clear that further study is required. The longitudinal impact of role modelling should also be evaluated through guided reflections on planned role modelling and through use of portfolios that span the training program.

## Electronic supplementary material

Below is the link to the electronic supplementary material.

Supplementary Material 1

## Data Availability

All data generated or analysed during this review are included in this published article.

## Abbreviations

PIF: Professional Identity Formation
SSR: Systematic Scoping Review
SEBA: Systematic Evidence Based Approach
PICOS: Population, Intervention, Comparison and Outcome
