# Supplementary material for: Role modelling in professional identity formation: a systematic scoping review
Source: BMC Med Educ. 2023 Mar 29;23:194. doi: 10.1186/s12909-023-04144-0 (PMC10052869; doi:10.1186/s12909-023-04144-0)
Supplement: Supplementary file 1 — Supplementary Material 1 [file 12909_2023_4144_MOESM1_ESM.pdf]

Appendix A. Tabulated Summaries

Author, Year	Study design	Intervention	Results	MERSQI	COREQ
Ahmadian Y. N. 2019	Qualitative	In this qualitative conventional content analysis, semi-structured individual interviews were conducted with 14 medical interns and 6 clinical professors. The participants were selected by purposive sampling. All interviews were recorded, transcribed, and analyzed. Trustworthiness, credibility, and confirmability of the data were confirmed.	Data analysis led to the emergence of a theme called role-modeling, and two subcategories: “advertent role-modeling” and “inadvertent role-modeling”. Advertent role-modeling included “influenced by the charismatic personality of professors”, “critique of faculty members’ communicative behaviors with patients”, and “observation of the faculty members’ performance”. Inadvertent role-modeling included “crystallization of human values in communication behaviors” and “compliance with hierarchical behavior”.	-	16
Armyanti I. 2020	Qualitative	The qualitative study using a transcendental phenomenology design was conducted at at Tanjungpura University, Indonesia, from December 2017 to February 2018, and comprised of 6 medical students from academic phase, 6 medical students from clinical phase, 8 medical teachers, 4 clinical teachers, 6 alumni and 5 programme managers. Data was collected through 5 focus group discussions and 5 in-depth interviews. Thematic analysis was applied to explore negative role mod- elling in the pre-clinical and clinical phase of the learning process. Data was analysed using the steps for coding and theo- risation method.	There were 30 respondents in five focus group discussions and 5 interviews were held with programme managers. There were three themes identified: medical teacher as a role model, process of role modelling, and nurturing medical pro- fessionalism. The presence of negative role modelling was evident in the discussions. Both positive and negative role mod- elling could influence the medical professionalism. Negative role modelling of medical teachers is a phenomenon often found in medical professionalism development.	-	15
Beanlands R. 2020	Qualitative	Recognizing the critical importance of mentorship for minority students, we developed a novel mentorship program for LGBTQ+ medical students at the Schulich School of Medicine and Dentistry (Schulich). To understand how mentors and mentees were impacted by Schulich’s LGBTQ+ mentorship program, we distributed online surveys to participants from the 2017-2018 and/or 2018-2019 academic years and performed a qualitative analysis. Our experience implementing this group and the results of this survey inform our	Online surveys were distributed via email, and seven (29%) medical students and four (50%) physicians provided a response. Medical students and mentors both identified personal and professional benefits to participation. Students felt that role-modelling by LGBTQ+ physicians provided reassurance about their capacity to achieve professional success, and they experienced a sense of belonging amongst peers and faculty (Table 1). For example, one student said, “The fact that other physicians have been out since the start of medical school and are still perfectly successful has also been reassuring.” Mentors identified the value of modelling	-	12

Appendix A. Tabulated Summaries

		recommendations to other medical schools on how best to implement similar programs.	success for gender and sexual minority students and gained new insight into the educational experience of minority learners. One mentor stated, "[...] I feel like I have been able to be a role model within my personal and professional life." Another noted, "I am more aware that my being open about my sexual orientation can help learners and create a more inclusive environment." Finally, physicians felt that participation fostered personal connection and led to networking with fellow mentors (Table 1).		
Bhatnagar V. 2020	Qualitative + Quantitative	To understand how mentorship impacts medical student decisions involving rotation choices, residency programs, field of practice, interest in research, and career trajectory. We hypothesized that effective mentor-mentee relationships would strongly impact medical students' decisions. Distributed to fourth-year osteopathic medical students at a single medical school, this study used a survey design to assess mentorship's impact on their aforementioned decisions.	Sixty-one students responded to this survey. Fifty-nine percent of respondents said they did not receive enough mentorship in medical school while 63.9% of respondents said their quality of mentorship was good/very good. Most survey respondents strongly agreed/agreed that the amount and quality of mentorship impacted their decisions involving rotation choices, residency programs, field of practice, and career trajectory. Qualitative data analysis led to the emergence of three themes: students pursuing primary care had positive mentorship experiences as compared to students pursuing non-primary care careers, female students stated they did not receive enough mentorship, and a majority of students cited the lack of formal mentorship as an area of improvement.	6	12
Chapman, L. 2020	Qualitative + Quantitative	Mixed methods approach. New Zealand registrars were invited to participate in anonymous survey regarding TMS experiences and learning. Focus groups explored skill acquisition and development more deeply.	A total of 121 registrars from 16 District Health Boards responded. Registrars supervise two juniors daily (range 0–4+). Fewer than 1:4 have formal training in TMS skills. Free text and focus group themes include: informal development by observing role models plus personal experience of giving and receiving TMS, inequitable access to development opportunities and formal training, barriers include workload and unsupportive learning cultures. Some registrars lack confidence in delivering TMS.	7	22

Appendix A. Tabulated Summaries

Lempp H. 2004	Qualitative	Design: Semistructured interviews with individual students. Setting: One medical school in the United Kingdom. Participants: 36 undergraduate medical students, across all stages of their training, selected by random and quota sampling, stratified by sex and ethnicity, with the whole medical school population as a sampling frame.	Students reported many examples of positive role models and effective, approachable teachers, with valued characteristics perceived according to traditional gendered stereotypes. They also described a hierarchical and competitive atmosphere in the medical school, in which haphazard instruction and teaching by humiliation occur, especially during the clinical training years.	-	12
Tomizawa Y. 2019	Commentary	-	-	-	-
Taylor C. A. 2009	Qualitative	OBJECTIVES: To understand the role and functions of mentoring and role-modeling in developing physician– leaders as experienced by aspiring and established physician–leaders. DESIGN: Qualitative design using a stratified purposeful sample and inductive analysis. APPROACH: Semi-structured interviews.	Twenty-five Cleveland Clinic faculty participated (14 established physician–leaders, 11 aspiring leaders). Three themes emerged: 1. Role modeling was differentiated as a valued experience separate from mentoring, with respondents describing the significant influence of purely observational learning and "watching leaders-in-action". 2. Many respondents favored a series of "strategic" interactions with various individuals about specific professional issues rather than traditional, longitudinal mentoring experiences. 3. Emotional and psychological support was considered the most valued type of interventional activity.	-	17
Thiedke C. 2004	Qualitative + Quantitative	First-year students completed a 14-item instrument regarding their com- munity-based physicians' behavior with patients. Responses were on a 5-point scale (1 = never, 5 = all of the time). Descriptive statistics were computed.	119 students completed the instrument (87% response rate). Students rated aspects of physicians' demeanor with patients highest (mean ranges 4.7–4.57). The lowest rated item was the physician's view of his or her professional role (M = 3.39), eliciting patients' ideas about illness and treatment (M = 3.55), and modeling inter- viewing techniques learned in class (M = 3.71).	9	8
Branch W. T. 2001	Qualitative	Despite repeated calls to emphasize the humanistic dimensions of care during medical education, these are few known techniques for effective teaching of humanism. We describe the barriers that inhibit humanistic teaching and suggest pragmatic teaching methods to overcome such barriers and teach humanistic care in clinical settings. We began	We found that barriers to teaching humanism largely consist of elements of the informal and hidden curricula in medical schools. We then defined methods to help teachers overcome these barriers. Specific methods fall into the 3 categories of taking advantage of seminal events, role modeling, and using active learning skills. We believe that formal courses and other well-motivated	-	8

Appendix A. Tabulated Summaries

		by asking participants at a conference on patient-physician communications sponsored by the American Academy on Physician and Patient in June 1998, "What can we do in the patient's presence to improve and teach the human dimensions of care? Please provide one or more examples of approaches you found to be effective." We augmented this information with suggestions from a number of colleagues in other settings. In a series of iterations, we analyzed all their suggestions to identify key teaching methods.	endeavors that take place away from patients fail to foster humanistic care. In contrast, we present pragmatic teaching methods that can be used in the fast-paced setting of the clinical environment.		
Lefkowitz A. 2020	Commentary	-	-	-	-
Hoskison K. 2019	Commentary	-	-	-	-
Hansen S. E. 2019	Qualitative	This qualitative study approached family medicine identity forma- tion from a social constructionist framework using evolved grounded theory. We performed a thematic analysis of focus groups conducted over 12 years with first-year residents (n=73). Then, utilizing a matrix analysis, articulations about professional identity were compared with structural components of the FMAHealth definition of the specialty. Three cohort groups (Preimplementation, Implementation, and Postimplementation) were defined to conduct a longitudinal comparison.	Six unique biosketches synthesizing the analyses emerged. Expan- sion in ability to articulate professional identity was evident not only across, but also within cohort groups. The Preimplementation cohort entered and left their first year identifying as relationship-centered generalists desiring guidance from role models. The Implementation learners used more FMAHealth language to describe their practice, later recognizing the potential it held for patient care. Similarly, the Postimplementation cohort entered with a broader view of family medicine and exited wondering how to help advance its reach.	-	16
Haque W. 2019	Qualitative	The authors in this qualitative study evaluated six 90-min focus groups of faculty members. The groups included 31 experienced and 19 inexperienced LC faculty members at the University of Texas Southwestern Medical School. After achieving excellent interrater reliability, transcriptions of the discussions were subjected to thematic analysis using ATLAS.ti software.	Five major themes emerged: (1) LC faculty characteristics/competency, (2) suggested faculty development methods, (3) factors outside the LC environment influencing student relationships, (4) student attributes influencing teaching techniques, and (5) measuring and improving history and physical skills. Faculty characteristics/competency subthemes included role-modeling, mentoring, and teaching competence. Suggested faculty development methods subthemes	-	14

Appendix A. Tabulated Summaries

			included assessing and giving feed- back to faculty, peer development, and learning from experts. Experienced LC faculty focused more attention on teaching competence and mentoring competence than inexperienced LC faculty.		
Herr K. D. 2020	Perspective	-	-	-	-
McKenzie S. 2020	Qualitative	Between 2015 and 2018, we conducted five focus groups, with a total of 36 volunteers, out of a possible 200 (18% Stage 3 (Year 3)) medical students. Each focus group session was audio recorded and transcribed verbatim. Participants were de-identified, and framework analysis used the theoretical frameworks of communities of practice, and workplace affordances to gain insight into their work-place learning experience during the first two months of their clinical rotation.	Thirty-six students out of 200 (18%) attended focus groups over a four-year period. The results are presented using the theoretical frameworks of community of practice and workplace affordances and presented as themes of: meaning, "learning as experience", practice, "learning as doing" community, "learning as belonging", and identity, "learning as becoming".	-	11
Elzubeir M. A. 2001	Quantitative + Qualitative	A 45-item self-administered questionnaire was sent to a sample (n ▯ 96, response rate 80%) consisting of three groups: (1) students in years 3±6 of the medical curriculum (n ▯ 66); (2) interns (n ▯ 17) and (3) residents (n ▯ 13). The questionnaire contained char- acteristics that participants might use to describe excellent role models, grouped under ®ve general headings: personality, clinical, research and teaching skills, and community service. Other characteristics mentioned by study subjects were qualitatively analysed using content analysis.	Personality and teaching and clinical skills were ranked as the top three factors, and research skills and community service as the least important factors by 79 (82%) respondents. Qualitative analysis of characteris- tics described by respondents for their role models yielded 21 characteristics. These were clustered into three main themes: role models as teacher, physician and person. The most frequently mentioned charac- teristics were personal characteristics such as positive, respectful attitudes toward patients and their families, and staff and colleagues; honesty; politeness; enthu- siasm; competence, and knowledge. Females rated nine personal characteristics signi®cantly higher than males (P < 0á05). Interns and residents valued teaching enthusiasm and competence signi®cantly more than students (P ▯ 0á01). Role models had a strong in¯u- ence on the specialty choice of 53 (55%) respondents.	8	8

Appendix A. Tabulated Summaries

Paukert J. L.<br>2000	Qualitative	On exit surveys in 1997 and 1998, students graduating from Baylor College of Medicine were asked to list clinical faculty who had ‘‘significantly and posi- tively influenced their clinical education’’ and to describe that influence. Using codes derived from Ullian’s earlier 1994 study regarding residents’ perceptions, the authors classified 1,153 written descriptions that expressed a sin- gle characteristic or teacher attribute into five roles: per- son, physician, supervisor, teacher, and unspecified (global). For each role, categories expressing similar char- acteristics were grouped into clusters. Although one author coded all descriptions, interrater reliability (.93) was determined by having an assistant code a set of ran- domly selected descriptions.	The medical students ranked characteristics of the teacher role highest. In contrast, Ullian’s residents ranked characteristics of the supervisor role highest.	-	7
Yazigi A.<br>2006	Quantitative	A structured and self-administered ques- tionnaire was sent to the cohort of interns (n ¼34) and residents (n ¼66) training in a Lebanese university hospital. The questionnaire contained pre- specified items related to professional and personal characteristics of positive and negative role models, as well as to the impact of these models on professional learning and career choices. Responders were asked to recognise and to rank- order the items associated to their identified models.	A total of 88 responders (97%) had positive role models and 87 responders (96%) had negative role models in their current training programme. Characteristics identified most frequently and ranked most highly by the trainees were related to clinical skills in positive role models and to inadequate humanistic and collaborative attitudes in negative models. Role modelling had a positive impact on the achievement of clinical skills for 55% of the responders, and on the acquisition of humanistic and collaborative attitudes for 30% of them. Thirty-eight per cent of the trainees were influenced by their role models in the choice of their specialities. Responses were generally comparable between levels of training and between medical and surgical specialities.	10	-
Janssen A. L.<br>2008	Perspective	-	-	-	-
Byszewski A.<br>2012	Quantitative + Qualitative	Survey on student perception of professionalism in general, the curriculum and learning environment at the University of Ottawa, and the perception of student behaviors, was developed by faculty and	The response rate was 45.6% (255 of 559 students) for all four years of the curriculum. 63% of the responses were from students in years 1 and 2 (preclerkship). Students identified role modeling as the single most important	7	9

Appendix A. Tabulated Summaries

		students and sent electronically to all University of Ottawa medical students. The survey included both quantitative items including an adapted Pritzker list and qualitative responses to eight open ended questions on professionalism at the Faculty of Medicine, University of Ottawa. All analyses were performed using SAS version 9.1 (SAS Institute Inc. Cary, NC, USA). Chi-square and Fischer's exact test (for cell count less than 5) were used to derive p-values for categorical variables by level of student learning.	aspect of professionalism. The strongest curricular recommendations included faculty-led case scenario sessions, enhancing interprofessional interactions and the creation of special awards to staff and students to "celebrate" professionalism. Current evaluation systems were considered least effective. The importance of role modeling and information on how to report lapses and breaches was highlighted in the answers to the open ended questions.		
Murakami M. 2009	Qualitative	Semi-structured interviews with thematic content analysis were implemented. Undergraduate year-5 students from a Japanese medical school at a Japanese teaching hospital were recruited. Interview were planned to last between 30 to 60 minutes each, over an 8-month period in 2007. The interviewees' perceptions concerning the quality of teaching in their bedside learning and related experiences were collected and analysed thematically.	Twenty five medical students (18 males and 7 females, mean age 25 years old) consented to participate in the interviews, and seven main themes emerged: "the perception of education as having a low priority," "the prevalence of positive/negative role models," "the persistence of hierarchy and exclusivity," "the existence of gender issues," "an overburdened medical knowledge," "human relationships with colleagues and medical team members," and "first experience from the practical wards and their patients."	-	16
Balmer D. 2007	Qualitative	We conducted a case study of 10 third-year pediatric residents and their 10 continuity clinic preceptors (CCP) in a community-based continuity clinic. Data were derived from 5 months (100 hours) of direct observation in clinic; semistruc- tured interviews with residents before and after observation; and semistructured interviews with CCPs after resident data were collected. Interview transcripts and notes from observation were inductively coded and thematically analyzed.	From the residents' perspective, role modeling was an implicit and intentional learning strategy that was linked to routine clinical practice in continuity clinic. Residents learned, through modeling their CCPs, "how to talk" and "how to think things through." Residents did not directly report modeling pro- fessional behavior. For residents, learning through modeling was not contingent on CCPs' awareness of being a role model.	-	21
Cruess R. L. 2006	Commentary	-	-	-	-
Joubert P. M. 2006	Qualitative	We used a qualitative method with symbolic interactionism and grounded theory as framework. Fourty two final-year students from the last cohort	There were no striking differences between the comments of the two groups. Students considered registrars to be the most influential role models in the	-	12

		following the traditional curriculum at the University of Pretoria in 2001, and 49 final years from the first cohort following the reformed curriculum in 2002 were recruited. Data were collected by applying focus groups, in-depth, individual interviews, as well as autobiographical sketches. Data were captured by means of audio tape recordings, transcripts of the tapes, researchers' field notes, and written accounts by students, and were analysed by using a general inductive approach.	clinical teaching context, followed by specialist consultants. Their idea of a good role model was a clinically and academically competent doctor that cared about patients, had good interpersonal skills, and who could inspire students. Students needed and appreciated good role models to help them to develop their own soft skills. They expected guidance and behavioural examples from clinical teachers. Although there were competent role models, the students were exposed to poor role models. Poor role models mainly affect students negatively. Students tend to imitate and perpetuate unacceptable behaviour. Furthermore, poor role models have a negative emotional effect on students and are detrimental to their moral and learning environment. Sometimes, poor role models have a paradoxical positive effect in the sense that they inform students how not to behave.		
Stalmeijer R. E. 2009	Qualitative	We explored students' experiences regarding the learning climate and whether the cognitive apprenticeship model fits students' experiences during clinical training. In focus group interviews, three groups of 6th-year medical students (N = 21) discussed vignettes rep- resenting the six teaching methods and the learning climate to explore the perceived occurrence of the teaching methods, related problems and possibilities for improvement.	The students had experienced all six teaching methods during their clerkships. Modelling, coaching, and articulation were predominant, while scaffolding, reflection, and exploration were mainly experienced during longer clerkships and with one clinical teacher. The main problem was variability in usage of the methods, which was attributed to teachers' lack of time and formal training. The students proposed several ways to improve the application of the teaching methods. The results suggest that the cognitive apprenticeship model is a useful model for teaching strategies in undergraduate clinical training and a valuable basis for evaluation, feedback, self-assessment and faculty development of clinical teachers.	-	19
Wear D. 2009	Qualitative	From June to November 2007, the authors conducted focus groups with attendings, residents, and students in one midwestern academic setting. The sessions were audiotaped, transcribed, and	All three groups offered a similar belief that the knowledge, skills, and values of the formal curriculum focused on building relationships. Similarly, all three suggested that elements of the informal and hidden curricula were expressed primarily as the values arising	-	15

		analyzed for themes surrounding the formal, informal, and hidden curricula	from attendings' role modeling, as the nature and amount of time attendings spend with patients, and as attendings' advice arising from experience and intuition versus "textbook learning." Whereas students and residents offered negative values arising from the informal and hidden curricula, attendings did not, offering instead the more positive values they intended to encourage through the informal and hidden curricula.		
Barnhoorn P. C. 2019	Perspective	-	-	-	-
Byszewski A. 2015	Quantitative + Qualitative	A literature review was performed and with the input of the AFMC(Association of Faculties of Medicine of Canada) Professionalism group, questionnaires were generated. An electronic survey was circulated to key leaders across the country at all the medical schools. In-depth telephone interviews were used to further explore themes, and a subsequent focus group was held to discuss challenges, particularly related to reporting and remediation.	The preponderance of formal professionalism teaching remains in the form of lectures and small group sessions in the preclinical years. Formal teaching declines significantly in the clerkship/clinical years. Evaluation is usually performed by a clinical supervisor, but OSCE, portfolio, and concern notes are increasingly used. Role modeling is heavily relied upon in clinical years, suggesting faculty training can help ensure clinical teachers recognize their influence on trainees. Formal remediation strategies are in place at most schools, and often involve essay writing, reflection exercises, or completion of learning modules about professionalism. Lack of clarity on what defines a lapse and fear of reprisal (for both trainees and faculty) limits reporting.	12	9
Hendelman W. 2014	Quantitative + Qualitative	An electronic survey, developed by faculty and medical students, was sent to all students with two email reminders. It included quantitative responses and some open-ended opportunities for comments. All analyses were performed with SAS version 9.1.	The response rate was 45.6% (255 of 559 students) for all four years of the medical school curriculum. Thirty six percent of students had witnessed or been part of an exemplary demonstration of professionalism; 64% responded that they had witnessed a lapse of professionalism. At the pre-clerkship level, the most frequent lapses involved students: arrogance (42.2%), impairment (24.2%), followed by cultural or religious insensitivity (20.5%). At the clerkship level of training, where students are exposed to real clinical situations, the lapses involved primarily faculty (including preceptor and	8	8

Appendix A. Tabulated Summaries

			clinician) or other staff; these included arrogance (55.3%), breach of confidentiality (28.3%), and cultural or religious insensitivity (26.6%); impairment involved mostly students (25.5%). These findings are analyzed from the perspective of role modeling by faculty and in the context of the learning environment.		
Irby D. M. 2016	Perspective	-	-	-	-
Sternszus R. 2016	Commentary	-	-	-	-
Cruess S. R. 2019	Perspective	-	-	-	-
Cruess R. L. 2015	Perspective	-	-	-	-
Sawatsky A. P. 2020	Qualitative	Using constructivist grounded theory, we conducted 23 semi-structured interviews with internal medicine residents at an academic medical centre. Interview transcripts were de-identified and processed through open coding and analytic memo writing. During data collection and analysis, we identified social cognitive theory (SCT), specifically reciprocal determinism, or the triadic and reciprocal rela- tionship between context, person and behaviour, as a useful theoretical lens through which to illuminate the relationship between autonomy and PIF. Using SCT to guide analysis, we organised themes, identified relationships amongst themes, and refined them through group discussion and constant comparison with new data.	Using constructivist grounded theory, we conducted 23 semi-structured interviews with internal medicine residents at an academic medical centre. Interview transcripts were de-identified and processed through open coding and analytic memo writing. During data collection and analysis, we identified social cognitive theory (SCT), specifically reciprocal determinism, or the triadic and reciprocal rela- tionship between context, person and behaviour, as a useful theoretical lens through which to illuminate the relationship between autonomy and PIF. Using SCT to guide analysis, we organised themes, identified relationships amongst themes, and refined them through group discussion and constant comparison with new data.	-	21
Mak-van der Vossen M. 2020	Perspective	-	-	-	-
Wald H. S. 2015	Perspective	-	-	-	-
Burgess A. 2015	Qualitative	The study was conducted with one cohort (n = 301) of students who had completed Year 1 of the	Students identified both positive and negative characteristics and behaviour displayed by their clinical	-	11

		Sydney Medical Program in 2013. A total of nine focus groups (n = 59) were conducted with medical students following completion of Year 1. Data were transcribed verbatim. Thematic analysis was used to code and categorise data into themes.	tutors. Characteristics and behaviour that students would like to emulate as medical practitioners in the future included: 1) Clinical attributes: a good knowledge base; articulate history taking skills; the ability to explain and demonstrate skills at the appropriate level for students; and empathy, respect and genuine compassion for patients. 2) Teaching skills: development of a rapport with students; provision of time towards the growth of students academically and professionally; provision of a positive learning environment; an understanding of the student curriculum and assessment requirements; immediate and useful feedback; and provision of patient interaction. 3) Personal qualities: respectful interprofessional staff interactions; preparedness for tutorials; demonstration of a passion for teaching; and demonstration of a passion for their career choice.		
McLean M. 2004	Qualitative + Quantitative	In 1997, the Faculty Board accepted a proposal to introduce Curriculum 2001, a self-directed learning (SDL) and problem- based (PBL) learning curriculum that replaced the tradi- tional, discipline-based curriculum in January 2001. In order to provide a reference with which to compare the evaluation of this new programme, a comprehensive survey of various aspects of students completing years 1–5 of the traditional six-year curriculum was undertaken. At the final year-end examinations in November 2000, students completed an anonymous questionnaire covering different aspects of their experiences in the faculty. One aspect canvassed student choice of role models and the qualities ascribed to an ideal role model. These results are presented on p. 135 of this issue (McLean, 2004). The study presently being reported summarizes the importance students ascribe to culture in their choice of role models.	- In a multicultural society, particularly one with a complex political and social history, culture may be an important issue in medical students' choice of role models.<br>- Identifying with a faculty role model from similar origins may be important for students to reaffirm their own culture, which is important as it may impact on their interaction with patients.<br>- Early and continuous diversity training for staff and students by appropriate individuals should be a main-stream academic activity to ensure acceptance and appreciation of other cultures.	6	6

Appendix A. Tabulated Summaries

Wright S. M. 2003	Qualitative	Between September and November 2000, in- depth semi-structured 30-minute interviews were con- ducted with 29 highly regarded role models, as judged by medical house officers at two large teaching hospitals in Baltimore, Maryland. Interview transcripts were in- dependently coded and compared for agreement. Content analysis identified several major categories of themes that were examined and conceptually organized.	The informants identified issues that relate to role modeling for diverse medical learners. Subcategories under the domain of similarity facilitates role modeling included learners prefer role models similar to them, role modeling is easier when the learner resembles the teacher, and minority physicians may be better role models for minority learners. Under the domain role modeling when physician–teachers and learners are different were the subcategories extra effort may be necessary, success promotes and inspires confidence, and role modeling across diversity is an achievable objective that should be pursued. The final domain, approaches to differences between physician–teachers and learners, encompassed embrace diversity, act as a consultant and refer when necessary, and minimize and disregard all differences.	-	18
Kenny N. P. 2003	Perspective	-	-	-	-
Haider S. I. 2020	Qualitative	The qualitative study using focus group discussions was held at Aga Khan University, Karachi from March to May 2018, and comprised medical students, residents and clinical teachers. Overall 11 focus group discussions were conducted till data saturation was achieved. Content analysis was used to analyse the data which was transcribed verbatim.	Of the 116 subjects, 60(51.7%) were medical students, 35(30.2%) were residents and 21(18%) were clinical teachers. Of the 11 focus group discussions, 4(36.5%) each were held with the students and the residents, while 3(27%) were held with the teachers. Five major themes that emerged from the study included definition of role models, attributes of role models, role modelling as a learnt behaviour, challenges in developing role models, and recommendations for developing positive role models. A number of attributes of positive and negative role models were identified by the participants. All the participants including students, residents and teachers appreciated the importance of role modelling in developing professionalism among health professionals and medical students. Factors hindering development and demonstration of positive role modelling were also identified and possible solutions were suggested.	-	24

Appendix A. Tabulated Summaries

Jones W. S. 2004	Qualitative	The authors discuss an innovative process, called Students'Clinical Ob- servations of Preceptors (SCOOP), which reverses the traditional direction of struc- tured observations. With written cues to focus their observations, students observe their preceptors, who intentionally model professionalism and communication during clinical encounters. Students and preceptors discuss the observed patient–physician interaction during postencounter sessions.	Most medical students rated the SCOOP process highly and reported professional behaviors they gained.	-	3
Benbassat J. 2014	Perspective	-	-	-	-
Passi V. 2013	Systematic review	A systematic search of electronic databases was conducted (PubMed, Psyc- Info, Embase, Education Research Complete, Web of Knowledge, ERIC and British Education Index) from January 1990 to February 2012. Data extraction was completed by two independent reviewers and included a quality assessment of each paper. A thematic analysis was conducted on all the included papers.	Thirty-nine studies fulfilled the inclusion criteria for the review. Six main themes emerged from the content of high and medium quality papers: 1) the attributes of positive doctor role models; 2) the personality profiles of positive role models; 3) the influence of positive role models on students' career choice; 4) the process of positive role modelling; 5) the influence of negative role modelling; 6) the influence of culture, diversity and gender in the choice of role model.	-	-
Passi V. 2016	Qualitative	This study used focus group interviews with 52 medical students, semi-structured interviews with 25 consultants and interviews after clinics with five consultants and five medical students. A qualitative methodology using the grounded theory inquiry approach of Strauss and Corbin was then used to generate an explanation of the process of modelling.	Role modelling is a process that involves conscious and subconscious elements and consists of an exposure phase followed by an evolution phase: The exposure phase involves demonstration of the attributes by the doctor role models. The evolution phase begins with observation of the role model, following which the modellee makes a judgement whether to trial the observed behaviours; when the decision to trial is reached, this then leads to a model-trialling cycle.	-	14
Wright S. M. 2001	Qualitative + Quantitative	Objective: To determine the values and attitudes which attending physicians try to pass on to residents in order to encourage their professional development.<br>Design: Cross-sectional study using a mailed survey.<br>Setting: Four university-affiliated teaching hospitals.	Of the 341 attending physicians who returned a completed questionnaire, 265 (78%) shared the single value or attitude they try to pass on to residents. The four main categories into which more than 95% of responses could be categorized were: (i) caring, (ii) respect, (iii) communication and (iv) integrity. There were no	5	8

Appendix A. Tabulated Summaries

		Subjects: Attending physicians with residency-level teaching responsibilities. Measurements: The self-reported single value or attitude that attending physicians try to pass on to house officers.	statistically signi®cant differences between the responses given by attending physicians who had been named as excellent role models and their colleagues who had not been so named.		
Wright S. M. 2002	Qualitative	We conducted 30-minute in-depth interviews with 29 highly regarded role models at 2 large teaching hospitals. We coded the transcripts indepen- dently, and compared our coding for agreement. Content analysis identified sev- eral major categories of themes.	The informants identified specific characteristics related to role modelling. Subcategories under the domain of personal qualities included interpersonal skills, a positive outlook, a commitment to excellence and growth, integrity and leadership. Under the domain of teaching, the subcategories were establishing rapport with learners, developing specific teaching philosophies and methods, and being committed to the growth of learners. Subjects thought there was some overlap between teaching and role modelling, but felt that the latter was more implicit and more encompassing. Being a strong clinician was regarded as neces- sary but not sufficient for being an exemplary physician role model. Perceived barriers to effective role modelling included being impatient and overly opinion- ated, being quiet, being overextended, and having difficulty remembering names and faces. Physician role models described role modeling consciousness, in that they specifically think about being role models when interacting with learners. Subjects believed that medical learners should emulate multiple role models.	-	11
Cruess S. R. 2008	Opinion	-	-	-	-
McLean M. 2004	Qualitative + Quantitative	Following completion of their final written examination, students were provided with an anonymous questionnaire covering various issues relating to their undergraduate medical studies. Of the 824 students writing the examinations, 97.5% of question- naires were recovered. One aspect of the questionnaire involved canvassing students with regard to their role models. Students were	Most students considering having a role model important. As students progressed, faculty role models were more likely to be selected. A parent (the mother, in particular) or parents were, however, most frequently identified as role models by all students. Attributes ascribed to parents as role models (caring, sympathetic, self-sacrificing) are similar to those considered desirable for professional role models in medicine.	7	6

Appendix A. Tabulated Summaries

		presented with the following description: ''A role model is someone you regard as a prototype on whom to model your functioning and behaviour. It might also be a person in a position to which you wish to aspire'', after which they were asked whether they had a role model (with explanation), if it was important for them to have a role model, and to describe an ideal role model. The objectives of the study were to ascertain where students sought role models, what character- istics they thought worthy of emulation, and if these role models varied between students in different years of their medical studies.			
Curry S. E. 2011	Qualitative	OBJECTIVES: Identifying and characterising exemplary, or positive, behaviours can be similarly valuable to both medical students and residents as tangible examples of behaviours to strive towards. The goal of the present research was to determine and thematically define the exemplary professional actions that medical students observe in the intense and patient-focused environment of the operating room (OR).<br>METHODS: Using qualitative methodology of content analysis and theme identification, we systematically documented the type of exemplary professional behaviours reported by medical students (n = 263) when observing health care teams on an anaesthesia rotation in the OR.	The analysis generated a taxonomy of exemplary OR behaviour that included six overarching themes (e.g. teamwork), 15 sub- themes (e.g. collegial) and numerous exemplars (e.g. showed mutual respect). These themes and sub-themes were then conceptually 'matched' – through the use of antonyms – to complement an existing framework focused on medical student reports of professional lapses witnessed during medical school.	-	11
Cote L. 2000	Qualitative	In the fall of 1997, 28 clinical teachers in fam- ily medicine and various medical and surgical specialties at Laval University Faculty of Medicine participated in individual semistructured interviews regarding their per- ceptions of the doctor–patient relationship and how it is taught. The interviews were conducted by a trained re- search assistant and the content of the interviews was coded by	The clinical teachers identified competencies associated with the doctor–patient relationship that dif- fered in complexity and specificity. Paramount among these competencies were the ability to conduct interviews effectively and politely, the ability to understand and in- volve the patient, and, in some cases, the ability to han- dle emotionally-charged situations. The clinical teachers tended to demand more of their students in doctor–pa-	-	16

Appendix A. Tabulated Summaries

		three independent observers, who then per- formed a qualitative analysis.	tient relationships than they did of themselves. Lack of time and a negative attitude toward the doctor–patient relationship, on the part of both teachers and students, were obstacles to teaching and learning this essential competency, even to the point of making it difficult for teachers to demonstrate and supervise these competencies during their daily clinical activities.		
Basco J. W. T.<br>2001	Quantitative	The 1998 graduating class of one medical school was surveyed about when and where they had made contact with their role models and whether they had made contact before or after making their specialty choices. Students also provided data about their demo- graphics, curriculum pathways (problem-based or tradi- tional), and specialty choices at matriculation and grad- uation.	Of the 89 graduating seniors who responded (62%), 21 had role models they had known prior to ma- triculation, 51 had encountered their role models in med- ical school, and 51 had met their role models before mak- ing their specialty choices. Of the 51 students who had encountered their role models in medical school, 33 (65%) had done so before making their specialty choices. The mean time from matriculation to meeting a role model was 24.9 6 11.6 months, and students on the problem-based learning pathway had met their role mod- els earlier than had students on the traditional pathway.	7.5	-
Lombarts K. M. J. M. H.<br>2010	Quantitative	Objectives: (i) To examine whether teaching qualities of faculty were associated with their being seen as a specialist role model by residents, and (ii) to investigate whether those associations differed across residency years and specialties.<br>Methods & Materials: Cross-sectional questionnaire survey amongst 549 Residents of 36 teaching programs in 15 hospitals in the Netherlands. The main outcome measure was faculty being seen as specialist role models by residents. Statistical analyses included (i) Pearson's correlation coefficients and (ii) multivariable logistic generalized estimating equations to assess the (adjusted) associations between each of five teaching qualities and 'being seen as a role model'.	407 residents completed a total of 4123 evaluations of 662 faculty. All teaching qualities were positively correlated with 'being seen as a role model' with correlation coefficients ranging from 0.49 for 'evaluation of residents' to 0.64 for 'learning climate' (P,0.001). Faculty most likely to be seen as good role models were those rated highly on 'feedback' (odds ratio 2.91, 95% CI: 2.41–3.51), 'a professional attitude towards residents' (OR 2.70, 95% CI: 2.34–3.10) and 'creating a positive learning climate' (OR 2.45, 95% CI: 1.97–3.04). Results did not seem to vary much across residency years. The relative strength of associations between teaching qualities and being seen as a role model were more distinct when comparing specialties.	8.5	-
Lynoe N.<br>2008	Quantitative	At the end of their first, fifth and last terms, 409 medical students from all six medical schools in Sweden participated in an attitude survey. The	Despite a low response rate at some schools, this study indicates that increased interest in medical ethics was related to encountering good physician role models, and	8	-

Appendix A. Tabulated Summaries

		questions focused on the students' experience of good and poor role models, attitudes towards medical ethics in general and perceived effects of the teaching of medical ethics.	decreased interest, to encountering poor role models. Physicians involved in the education of medical students seem to teach medical ethics as role models even when ethics is not on the schedule.		
Matthews C. 2000	Qualitative + Quantitative	Method: Using semi-structured interviews of faculty and a questionnaire based on the issues arising from the interviews, faculty members' recollections of their medical teachers' behaviours were compared with resi- dents' current perceptions of the same teaching behaviours.<br>Setting: Department of Family Medicine, King Fahad National Guard Hospital, Riyadh, Saudi Arabia.<br>Subjects: Faculty and residents.	The four best-remembered teacher behaviours were: positive behaviour towards patients, negative behaviour towards junior colleagues, effective presen- tation of subject content and encouragement to par- ticipate in patient care. The residents perceived positive behaviour towards patients, positive behaviour towards junior colleagues, suboptimal skills of subject content presentation, and insuf®cient encouragement for trainees to actively participate in patient management. Although faculty retained many unhappy memories of teacher behaviour, it was encouraging that there was no evidence of perpetuation of the negatively perceived behaviours which provoked them.	6	17
Park J. 2010	Qualitative	The authors conducted 34 semistructured interviews of individual surgery residents and faculty members at two academic institutions from 2004 to 2006. Interviews consisted of open-ended questions on how the participants learned professionalism and what they perceived as challenges to learning professionalism. Two researchers analyzed the interview transcripts for emergent themes by using a grounded-theory approach.	Faculty members' and residents' perceptions of how they learned professionalism reflected four major themes: (1) personal values and upbringing, including premedical education experiences, (2) learning by example from professional role models, (3) the structure of the surgery residency, and (4) formal instruction on professionalism. Of these, role modeling was the dominant theme: Participants identified observation, reflection, and reinforcement as playing key roles in their learning from role models and in distinguishing the sometimes blurred boundary between positive and negative role models.	-	18
Ravindra P. 2011	Qualitative + Quantitative	Background: Positive encounters with surgeons have previously been shown to influence perceptions of surgical careers. Despite this, negative perceptions persist. We investigated whether identifying role models in surgery influences career choice and defined the ideal qualities of a surgical role model as perceived by newly qualified doctors.	Questionnaires were returned by 208 of 320 graduates (65%). Median age was 24 years (range = 23–51); 63% female, 37% male; 71% standard undergrad- uate course, 28% graduate-entry course. Overall, 131 respondents (63%) felt they were able to identify a surgical role model; there were no statistically significant differences between gender or course type. There was a significant dif- ference between identification of a surgical role model and	6.5	9

Appendix A. Tabulated Summaries

		Methods: A 36-item questionnaire was distributed to newly qualified graduates from a large UK medical school. Results were analysed using GraphPad Prism 5.00.	interest in pursuing surgical careers (P = 0.0006), with 41% of those who identified a role model interested compared with 17% of those who did not. Overall, 564 key qualities for a surgical role model were suggested by respondents. These were grouped by theme, with common attributes including good teacher, enthusiastic, and approachable.		
Shortell C. K. 2008	Quantitative	The purpose of this study was to investigate whether selective lifestyle, mentorship, and associated job considerations were more commonly identified by female vascular surgeons during their decision- making process for their specialty. The study used a survey sent to vascular surgery residents and fellows. Questions were related to mentorship, lifestyle, and job characteristics and were analyzed using a series of bivariate comparisons.	The findings suggest that both men and women rarely report a female mentor in medical school, and that female vascular surgeons were less likely than others to have children. As in other studies, we found that females lack female role models during medical school. With respect to career choice, no lifestyle or job characteristics were statistically different between female vascular surgeons and male counterparts.	9	-
Wear D. 2006	Qualitative	The authors conducted five voluntary focus groups over a three-month period with 58 third- and fourth-year medical students at the Northeastern Ohio Universities College of Medicine in 2005. After transcribing the taped interviews, the authors analyzed the data using qualitative methods and identified themes found across groups.	The categories that emerged from the data were (1) categories of patients who are objects of humor, including those deemed “fair game” due to obesity or other conditions perceived as preventable or self-inflicted; (2) locations for humor; (3) the “humor game,” including student, resident, and faculty interaction and initiation of humor; (4) not-funny humor; and (5) motives for humor, including coping and stress relief.	-	15
White C. B. 2009	Qualitative	The authors solicited members of the first cohort to complete the FCE (the class of 2007) to participate in this focus- group-based study halfway through the third year. They explored the influence of the FCE on students’ experiences in the third-year clerkships, and how conflicts between the two learning experiences shaped these students’ values and behaviors.	Students reported that during clerkships they experienced strong feelings of powerlessness and conflict between what they had learned about patient- centered care in the first two years and what they saw role modeled in the third year. Based on students’ comments, the authors categorized students into one of three groups: those whose patient- centered values were maintained, compromised, or transformed.	-	19
Weismann P. F. 2006	Qualitative	Using an observational, qualitative methodology, the authors studied 12 clinical faculty identified by the medical residents enrolled from 2003 to 2004	Clinical teachers taught primarily by role modeling. Although they were highly aware of their significance as role models, they did not typically address the human	-	9

Appendix A. Tabulated Summaries

		as excellent teachers of humanistic care on the inpatient medical services at four medical universities in the United States (University of Minnesota Medical School, Emory University, University of Rochester School of Medicine, and Baylor College of Medicine). Observations were conducted by the authors using standardized field notes. After each encounter, the authors debriefed patients, learners (residents and medical students), and the teaching physicians in semistructured interviews.	dimensions of care overtly. Despite the common themes of role modeling identified, each clinical teacher exhibited unique teaching strategies. These clinical teachers identified self- reflection as the primary method by which they developed and refined their teaching strategies.		
Wyber R. 2007	Qualitative	In November 2004, interviews were held with a convenience sample of six male and six female general practitioners (GPs) from the urban Dunedin area who completed their house surgery years in New Zealand and were provisionally registered at least ten years ago (Group 1).16 GPs are in an ideal position to reflect on their house office years with limited subsequent exposure to hospital culture. After training and a practise interview with the second author, the medical student first author conducted the interviews. The semi-structured interview, lasting about half an hour, was conducted in person at practice rooms around the city. The interviews began with the volunteers providing an example of 'an experience, person or incident' that they considered represented positive, and then negative, role modelling during their house officer years. The third part of the interview covered reflection and consciousness of role modelling during and after the events. In December 2005 a convenience sample of 3 male, and 10 female, current house officers at Dunedin Public Hospital (Group 2) participated in interviews using the same probes.17 These participants were PGY1 and PGY2 doctors with standard house officer	The interviews revealed three broad relationships that house officers consider important for identifying their role models: the relationship between house officer and the model; the model's relationship with patients; and the model's relationship with medicine. Clinical skills are excluded from this discussion because they are generally a poor demarcation between positive and negative role models.	-	17

Appendix A. Tabulated Summaries

		rotations in medicine, surgery, and specialties. The interviews were held in person, most at the Dunedin Public Hospital while the house officers were on duty. In general, the second round of interviews was shorter and slightly less structured than the first. All interviews were taped, independently transcribed, and underwent multiple readings by the first author to identify key themes. These themes, and samples from the transcripts, were regularly discussed with the second author throughout coding and analysis. Themes are illustrated with verbatim quotes in the results section; numbers in parenthesis refer to individual respondents.			
Berman L. 2008	Qualitative + Quantitative	Medical students completed a survey at the end of the surgical clerkship assessing characteristics of the clerkship experience and students' level of interest in pursuing a career in surgery. The survey also included open-ended questions about students' reasons for having increased or decreased interest in surgery, which were systematically analyzed to complement quantitative findings.	Students who sutured (p =0.001), drove the camera (p =0.01), stated that they felt involved in the operating room (p =0.009), and saw residents (p 0.03) and attendings (p =0.0003) as positive role models were more likely to be interested in surgery. After adjusting for covariates, students who sutured in the operating room were 4.8 times as likely to be interested in surgery (95% CI, 1.5 to 14.9) and students who drove the camera were 7.2 times as likely to be interested in surgery (95% CI, 1.1 to 46.8).	8	12
Paice E. 2002	Perspective	-	-	-	-
Abaza M. M. 2018	Commentary	-	-	-	-
Abdul-Khaliq I. 2020	Letter	-	-	-	-
Adkoli B. V. 2011	Qualitative	We adopted qualitative approach including 10 focus group discussions. The proceedings were tape-recorded, transcribed, and analyzed independently by two researchers.	The respondents admitted that that they were deficient in the acquisition of professional values. According to them, professionalism was not taught or assessed. They followed ''hidden curriculum''. They considered very few teachers as positive role models. The deficiencies could be attributed to negative role modeling by the faculty or	-	14

Appendix A. Tabulated Summaries

			deficiencies in the curriculum such as lack of rich clinical experiences, limited interaction with health team, and absence of feedback besides organizational issues.		
Agarwal R. 2010	Quantitative	A Web-based survey was sent to the 35 residents in the 2007 Siemens AUR Radiology Resident Academic Development (SARRAD) program and to all other residents at their institutions. The survey contained questions regarding experiences with and desirable characteristics of role models, as well as the influence of role models in career decisions. Chi-square, Fisher's exact, and Mann-Whitney tests were used to assess associations between responses and expressed career choice.	Thirty of 35 SARRAD participants (85%) plus 103 non-SARRAD participants responded. Only 46% felt that there were enough role models at their institutions and 56% that there were sufficient role models in academic radiology. More than two-thirds of residents surveyed stated that they would be more likely to stay in academic radiology if there were more role models. The most desired characteristics of role models included availability, enthusiasm, integrity, and a positive attitude toward residents. Residents stating that they would choose academic careers were more likely to be research track (P.0001), have more publications (P.01), be less concerned with salary (P.003), and be less concerned about politics (P.047). Level of debt was not different between residents planning to choose academic careers and those with other career plans (P.80).	8	-
Amalba A. 2016	Qualitative + Quantitative	A cross-sectional survey was conducted by means of a questionnaire among medical students to explore the char- acteristics of positive and negative role models during COBES. Associations between gender, choice of specialty, and practice location were assessed using the chi-square test. All qualitative data analysis was performed using the principles of primary, secondary, and tertiary coding.	The majority of the students indicated that role modeling during COBES will affect their choice of specialty and prac- tice location with a significant gender difference in terms of practice location ($p < 0.005$). Qualitative data supported the finding that positive role modeling during COBES may influence graduates willingness to work in rural area.	9	11
Arah O. A. 2012	Qualitative + Quantitative	We carried out a cross-sectional survey within a longitudinal study of the System for Evaluation of Teaching Qualities (SETQ) of clinical teachers. The study sample included 889 residents and 1014 faculty members in 61 teaching programmes spanning 22 specialties in 20 hospitals in the Netherlands. Main outcome measures included residents' (i) global and (ii) specific ratings of faculty	In total, 690 residents (77.6%) com- pleted 6485 evaluations of 962 faculty members, 848 (83.6%) of whom also self-evaluated. More recently certified faculty members, those who had attended a teacher training programme, and those who spent more time teaching than seeing patients or conducting research were more likely to score highly on most teaching qualities. However, faculty members who had undergone teacher training	12	9

Appendix A. Tabulated Summaries

		member teaching qualities, and (iii) global ratings of faculty members as role-model specialists. Statistical analysis was conducted using adjusted multivar- iable logistic generalised estimating equations.	were less likely to be seen as role models (odds ratio [OR] 0.72, 95% confidence interval [CI] 0.59–0.88). In addition, faculty members were evaluated slightly higher by male than female residents on core teaching domains and overall teaching quality, but were less likely to be seen as role models by male residents (OR 0.80, 95% CI 0.67–0.97). Lastly, faculty members had higher odds of receiving top scores in specific teaching domains from residents in the first 4 years of residency and were less likely to be considered as role models by more senior residents.		
Asghari F. 2011	Quantitative	In this descriptive study, 218 interns in surgery and internal medicine wards at four teaching hospitals of Tehran University of Medical Sciences were enrolled during the first semester of the 2007/2008 academic year. Each intern completed one questionnaire for faculty and one for residents in their ward.	The questionnaire was completed by 150 students (68.8%) for faculty and by 139 students (63.7%) for residents. In terms of overall aspects of professionalism, the mean (6SD) score on observing professionalism was 52.9610 and 49.2667.9 points out of 70 for faculty and residents, respectively. Students agreed that responsibility was observed better than other aspects of professionalism (119/150 (79.3%) and 115/139 (82.8%) students stated it was often or always observed by faculty and residents, respectively). In both groups, commitment to honour and integrity and excellence was less compared with other areas.	10.5	-
Azmand S. 2018	Qualitative	Semi-structured and in-depth interviews were used to collect medical students' experiences and viewpoints, which were then analyzed through simple content analysis and the codes and categories were extracted. Finally, themes were derived as the central organizing concepts.	Saturation occurred after 17 interviews. Seven main themes were extracted as the working components of hidden curriculum regarding professionalism in the setting: ‘convenient patients’, ‘evaluate me’, ‘trust as the base of team interactions’, ‘perceiving encouragement’, ‘relationship satisfaction and authenticity’, ‘workload and students’ well-being’ and ‘role modeling at the heart of professionalism’.	-	16
Baernstein A. 2009	Qualitative	Individual semistructured interviews were conducted with 56 students completing the preclinical curriculum at the University of Washington School of Medicine in 2004 and 2005.	Students identified role modeling as an important modality for learning professionalism, even during their preclinical years. Role models included classroom faculty and peers, in addition to physicians in clinical settings. Small- group discussions and lectures helped some	-	16

Appendix A. Tabulated Summaries

		Interviews were recorded, transcribed, and analyzed using qualitative methods.	students identify and analyze the professional behaviors they observed, but they elicited negative responses from others. Students believed their professionalism derived from values, upbringing, and experiences prior to medical school. Some students reflected on their evolving professionalism while working directly with patients.		
Bahman-Bijari B.<br>2016	Quantitative	Quantitative empirical data were gathered using a self-administered questionnaire by graduating students in medical, dentistry, and pharmacy schools at Kerman University of Medical Sciences. A total of 3 graduating cohorts, comprising about 220 students, were selected for this study. Surveys were distributed during January-March 2013.	In total, 183 students participated in the study. Altogether, students considered 504 and 473 academic staff as positive and negative role models (PRMs and NRMs), respectively. Women were considered more negatively than men (mean scores: -12.13 vs. -11.6, p=0.04). While clini-cians were considered more positively than basic scientists (mean scores: 12.65 vs. 10.67, p=0.001), dentists received higher positive scores than physicians or pharmacists (average scores: 13.27 vs. 12.99 and 9.82). There was a significant relationship between the personality of the students and the overall characteristics of their perceived role models (β for PRMs=0.35, p<0.0001; and β for NRMs= 0.20, p= 0.039).	9	-
Bahman-Bijari B.<br>2017	Quantitative	In an analytical cross-sectional study, a structured and self-developed questionnaire was completed by 185 medical students at educational hospitals of Kerman University of Medical Sciences during April and May 2015. Participants were selected using convenience sampling method. For data analysis, we used descriptive and inferential statistics. SPSS software version 16 was used as needed.	In total, 90 medical students (48.7%), 65 interns (35.1%), and 30 residents (16.2%) participated in this study. Male respondents (n=75) comprised 40.5% and female respondents (n=110) 59.5% of the study sample. The three most important roles of a clinical teacher were organizer role (99.7), teacher role (101.7), and supporter role (109.5) for students, interns, and residents respectively. On the other hand, supporter role (85.4), communicator role (86.4) and organizer role (83.4) were ranked as the least important for students, interns, and residents respectively. There was no significant association among the three batches and the roles of a clinical teacher (p>0.05). Conversely, Females rated the roles of a clinical teacher significantly higher than males (p<0.05).	5	-
Baker M.<br>2011	Qualitative	Ten UCSF medical students were interviewed at three time points (second, third, and fourth years of	Students described varying steps in their professional development from their second to fourth years of school,	-	11

		school). Interviews focused on students' learning and development regarding end-of-life care (EOLC).	including feeling confused about the definition of professionalism and integrating their personal and professional identities. In addition to professional development, four other themes contributed to the development of medical student understanding of how to provide EOLC as a professional: (1) curricular discordance, (2) role models, (3) the tightrope between trained versus human reactions, and (4) ethical dilemmas.		
Bakken L. L. 2005	Quantitative	A 35-item clinical research self-efficacy questionnaire was administered to 251 health care professionals who attended programs at the University of Wisconsin- Madison from 2002–2004. Three questions were included to determine the sex, role, and qualities of the expert that are envisioned by participants. Frequency distributions were computed for each response and variables were compared by gender using chi-square analysis and Fisher exact test.	Ninety-five physicians-in-training and junior faculty physicians responded to the questionnaire. Seventy-one percent of female and 95% of male respondents reported their envisioned experts to be male. The most frequently reported role of the envisioned expert was that of a mentor who was a faculty member in the respondent's own department (72% women, 60% men). The three most frequently reported qualities of the envisioned expert were "multiple publications," "scientific knowledge," and "supportiveness." However, women more frequently reported "communication skills" and "problemsolving abilities" than did men. This difference was statistically significant and largely due to the frequency of qualities selected by women whose envisioned expert was female.	7	-
Bazrafkan L. 2019	Quantitative + Qualitative	In the quantitative part, data were collected using a questionnaire with 24 items. The research population included medical students who were in their clinical period between May 2017 and December 2018 at Shiraz University of Medical Sciences (n = 750). A total of 282 questionnaires were completed by these students, and in the qualitative part, 26 semi-structured interviews were conducted with them.	The most important components of role modeling for students included: individual characteristics, clinical skills and competence, teaching skills and professionalism, in that order. The qualitative analysis confirmed the results of the quantitative analysis. The findings showed that the characteristics of a negative role model can also be classified in four main components. The results demonstrated that 46.8% of the students identified one or more medical teachers as negative models.	11	12
Birden H. 2013	Systematic review	Eligible studies included any articles published between 1999 and 2009 inclusive. We reviewed papers presenting viewpoints and opinions as well as empirical research. We performed a comparative and thematic synthesis on all papers meeting	We identified 217 papers on how to teach professionalism. Of these, we determined 43 to be best evidence. Few studies provided comprehensive evaluation or assessment data demonstrating success. As yet, there has not emerged a unifying theoretical or	-	-

Appendix A. Tabulated Summaries

		inclusion criteria in order to capture the best available evidence on how to teach professionalism.	practical model to integrate the teaching of professionalism into the medical curriculum.		
Boerebach B. C. M. 2012	Quantitative	In a prospective multicenter multispecialty study of faculty's teaching performance, we used web-based questionnaires to gather empirical data from residents. The main outcome measures were the different typologies of role modelling. The predictors were faculty's overall teaching performance and faculty's teaching performance on specific domains of teaching. The data were analyzed using multilevel regression equations.	In total 219 (69% response rate) residents filled out 2111 questionnaires about 423 (96% response rate) faculty. Faculty's overall teaching performance influenced all role model typologies (OR: from 8.0 to 166.2). For the specific domains of teaching, overall, all three role model typologies were strongly associated with ''professional attitude towards residents'' (OR: 3.28 for teacher/supervisor, 2.72 for physician and 7.20 for the person role). Further, the teacher/supervisor role was strongly associated with ''feedback'' and ''learning climate'' (OR: 3.23 and 2.70). However, the associations of the specific domains of teaching with faculty's role modelling varied widely across specialties.	11.5	-
Boerebach B. C. M. 2013	Commentary	This study revisits a previously published study about the influence of faculty's teaching performance on their role modeling (as teacher-supervisor, physician and person). We drew eight directed acyclic graphs (DAGs) to visually represent different plausible causal relationships between the variables under study. These DAGs were subsequently translated into corresponding statistical models, and regression analyses were performed to estimate the associations between teaching performance and role modeling.	The different causal models were compatible with major differences in the magnitude of the relationship between faculty's teaching performance and their role modeling. Odds ratios for the associations between teaching performance and the three role model types ranged from 31.1 to 73.6 for the teacher-supervisor role, from 3.7 to 15.5 for the physician role, and from 2.8 to 13.8 for the person role.	-	-
Brisette. M. D. 2017	Quantitative	Surveys were sent to all College of American Pathologists junior members and all pathology residency program directors, and responses were compared.	Although no single behavior received the same professionalism rating among residents and program directors, both groups identified the same behaviors as being the most unprofessional: posting identifiable patient information or case images to social media, making a disparaging comment about a physician colleague or member of the support staff on social media or in a public hospital space, and missing work without reporting the time off. Faculty were observed displaying most of these behaviors as often or more often than	7	-

Appendix A. Tabulated Summaries

			residents by both groups. The most common means to teach professionalism in pathology residencies is providing feedback as situations arise and teaching by example. Age differences were found within each group and between groups for observed behaviors and attitudes.		
Brown M. E. L. 2020	Qualitative	Semi-structured focus group interviews were conducted with 39 students from one UK medical school. Fourteen faculty were interviewed individually to triangulate data. Data were analysed using constructivist thematic analysis, informed by grounded theory convention.	The presence of the hidden curriculum was clearly demonstrated, acting through role modelling, organizational culture, stereotyping and professional dress. Mentioned frequently were the influences of the hidden curriculum on student professionalism and identity development. Professionalism was perceived as being negatively impacted by the hidden curriculum and seen as an imposition from senior faculty to control students. Students believe medical identity formation begins prior to medical school, in a process known as "anticipatory socialization", a previously unstudied identity transition. Students felt covert institutional agendas negatively impacted their identity, pushing them further from the identity their institution was encouraging them to acquire. Key messages for educators include the need to explore the hidden curriculum through discussion with students.	-	13
Burgess A. 2016	Qualitative + quantitative	The study was conducted with one cohort (n = 301) of students who had completed year 1 of the medical programme in 2013. All students were asked to complete a questionnaire regarding the ideal attributes of a good role model in a clinical tutor. The questionnaire consisted of seven closed items and one open- ended question.	The response rate to the questionnaire was 265/301 (88%). Although students found all three key areas important in a good role model, students emphasised the importance of excellence in teaching skills. Specifically, students see good role models as being able to provide a constructive learning environment, a good understanding of the curriculum and an ability to cater to the learning needs of all students.	8	1
Collier A. 2013	Quantitative	Data were collected on the cohort starting work in 2010 until applying to a specialty training program 16 months later. Total hours of psychiatry teaching was compared with the number choosing a residency in the same specialty.	A total of 19 hospitals in northwest England provided teaching programs for their interns and first-year residents (U.K. foundation doctors); 15 provided information on doctors' later specialty choice. Only 2.3% of teaching was dedicated to psychiatry. Doctors led a	9.5	-

Appendix A. Tabulated Summaries

			higher proportion of medicine or surgery sessions (63%) than those on psychiatry (48%). Provision of psychiatry teaching was associated with entering psychiatry residency.		
Cote. L. 2014	Qualitative	In 2010, the authors conducted a descriptive qualitative study with preceptors in medical, surgical, and laboratory specialties who supervised residents on a regular basis at the Université Laval Faculty of Medicine (Québec, Canada). Respondents participated in semistructured, individual interviews. An inductive thematic analysis of interview transcripts was conducted using triangulation.	Most participants highlighted the importance of role modeling to support residents' development of the CanMEDS competencies, particularly communication, collaboration, and professionalism, which preceptors perceived as "less scientific" and the most difficult to teach. Although most participants reported using an implicit, unstructured role modeling process, some described more explicit strategies. Eight types of educational challenges in role modeling the CanMEDS competencies were identified, including encouraging reflective practice, understanding the competencies and their importance in one's specialty, and being aware of one's strengths and weaknesses as a clinical teacher.	-	11
Cruess R. L. 2018	Perspective	-	-	-	-
Doja A. 2016	Qualitative	Focus groups were held with undergraduate and postgraduate learners and faculty to explore knowledge and perceptions relating to the hidden and informal curricula. Thematic analysis was conducted both inductively by research team members and deductively using questions structured by the existing literature.	Participants highlighted several themes related to the presence of the hidden and informal curricula in medical training and practice, including: the privileging of some specialties over others; the reinforcement of hierarchies within medicine; and a culture of tolerance towards unprofessional behaviors. Participants acknowledged the importance of role modeling in the development of professional identities and discussed the deterioration in idealism that occurs.	-	15
Egnew T. R. 2011	Qualitative	Qualitative data from focus groups and long interviews were coded by the authors through an iterative dialogic process. Participants were 15 faculty and 35 medical students in clinical training in a New Zealand medical school.	Teaching of doctor-patient relationship skills was highly variable, rarely explicit, and heavily dependent on role modeling. Students noted variable focus on relational skills between rotations, incongruity between preclinical training and the behaviors observed in clinical environments, and a need to discern which relational skills were facilitative. Role models who transparently	-	9

Appendix A. Tabulated Summaries

			shared their personal experiences of doctoring were more effective in helping students learn relationship skills.		
Essers G.<br>2012	Quantitative	In a cross-sectional study, data were collected about physicians' communication performance as perceived by students. Students filled out a questionnaire in four different clerkships in their fourth and fifth year.	Just over half of the students reported communication similar to formal training. This was especially true for students in the later clerkships (paediatrics and primary care). Good examples were seen in providing information corresponding to patients' needs and in shared decision making, although students often noted that in fact the doctor made the decision. Bad examples were observed in exploring cognitions and emotions, and in providing information meeting patient's pace.	8	-
Feraco A. M.<br>2016	Narrative review	-	-	-	-
Finn G.<br>2010	Qualitative	Seventy-two undergraduate students from two UK medical schools participated in 13 semi-structured focus groups. Focus groups, carried out until thematic saturation occurred, were recorded and transcribed verbatim. Data were analysed and coded using NVivo 8, using a grounded theory approach with constant comparison.	From the analysis, seven themes regarding professionalism emerged: the context of professionalism; role-modelling; scrutiny of behaviour; professional identity; 'switching on' professionalism; leniency (for students with regard to professional standards), and sacrifice (of freedom as an individual). Students regarded professionalism as being relevant in three contexts: the clinical, the university and the virtual. Students called for leniency during their undergraduate course, opposing the guidance from Good Medical Practice. Unique findings were the impact of clothing and the online social networking site Facebook on professional behaviour and identity. Changing clothing was described as a mechanism by which students 'switch on' their professional identity. Students perceived society to be struggling with the distinction between doctors as individuals and professionals. This extended to the students' online identities on Facebook. Institutions' expectations of high standards of professionalism were associated with a feeling of sacrifice by students caused by the perception of constantly 'being watched'; this perception was coupled with resentment of this intrusion. Students	-	19

Appendix A. Tabulated Summaries

			described the significant impact that role-modelling had on their professional attitudes.		
Foster K. 2016	Qualitative	Personal Interview Narratives were derived from the stories told by twelve senior doctors as they recalled accounts of people and events from the past that shaped their notions of being a doctor. Narrative inquiry methodology was used to explore and analyse video recording and transcript data from interviews.	Role models were frequently characterised as heroic, or villainous depending on whether they were perceived as good or bad influences respectively. The degree of sophistication in participants' characterisations appeared to correspond with the stage of life of the participant at the time of the encounter. Heroes were characterised as attractive, altruistic, caring and clever, often in exaggerated terms. Conversely, villains were typically characterised as direct or covert bullies. Everyday events were surprisingly powerful, emotionally charged and persisted in participants' memories much longer than expected. In particular, unresolved emotions dating from encounters where bullying behaviour had been witnessed or experienced were still apparent decades after the event.	-	16
Haghdoost A. A. 2006	Quantitative	Staff and students were questioned about the characteristics of their colleagues and lecturers, respectively. They were asked about 15 characteristics under four headings: personality, teaching skill, group working and overall performance as a role model. Associations between lecturers' characteristics were explored using Pearson correlation and characteristics were allocated into groups by partition cluster method. In addition, predictors of being a valuable lecturer were assessed using logistic regression analysis.	Based on staff responses, the strongest association observed was between honesty and being respectful ($r = 0.93$, $p < 0.0001$). Based on student responses, the strongest association observed was between being professional and honesty ($r = 0.98$, $p < 0.0001$). None of the correlations between student and staff perceptions were significant for any characteristic. Two groups were recognized among the characteristics. group one contained those characteristics which were related to the lecturer's activity; while the second group contained characteristics that were related to the personality or teaching performance of the lecturer. The predictors of lecturer as 'role model' (i.e., perceptions of students) consisted mostly of characteristics from the first group, while the predictors of a 'role model' by fellow academic staff consisted of characteristics that were in both groups.	6	-
Haider S. I. 2016	Quantitative	A 32 item questionnaire was developed and self-administered to undergraduate medical students. Participants rated the characteristics on a three	A total of 349 (65.23%) distributed questionnaires were returned. The highest ranked themes were teaching and facilitating learning, patient care and continuing	8.5	-

Appendix A. Tabulated Summaries

		point scale (0 = not important, 1 = mildly important, 2 = very important). One way ANOVA and student's t-test were used to compare the groups.	professional development followed by communication and professionalism. Safe environment and guiding personal and professional development was indicated least important. Differences were also observed between scores obtained by males and females.		
Harris G. D. 2004	Commentary	-	-	-	-
Healy N. A. 2012	Systematic review	A comprehensive PubMed search of the literature on the subject of role models and mentors was performed using the following keywords: “Mentors,” “Mentorship,” and “Role Models” alone and in conjunction with the words “medicine” and “surgery.”	This article defines the terms role model and mentor and highlights the differences between these. It identifies the importance of early intervention in medical students’ careers by surgeons and the possibility of junior doctors acting as mentors. Formal mentoring programs appear to be associated with greater satisfaction among surgical trainees regarding mentorship. In addition, this review serves to show the potential approaches to developing mentorship and role models in surgery.	-	-
Healy N. A. 2013	Quantitative	A questionnaire was distributed to senior undergraduate medical students in 1 medical school, and postgraduate surgical trainees (members of the Association of Surgeons in Training (ASIT) in the UK and Ireland. The survey included questions about the availability of mentors and role models and explored mentorship process.	A total of 163 medical students and 216 surgical trainees completed the questionnaire. While most medical students did not have a mentor, 52% (n 104) of trainees reported having a surgical mentor. In both cases, mentoring was ill-structured and informal. While most medical students expressed a preference for a formal mentoring program, only 38% of surgical trainees expressed a preference for a more formal approach. Experiences of negative surgical role models were a pervasive feature for both medical student and surgical trainee respondents.	7.5	-
Holden J. 2020	Commentary	-	-	-	-
Holt G. R. 2008	Commentary	-	-	-	-
Horsburgh J. 2018	Qualitative	To gain insight into medical students’ and clinical teachers’ understanding of learning through role modelling, a qualitative, interpretative methodology was adopted, using one-to-one semi-structured interviews. Six final year medical	Students could identify ways in which they learnt from role models but acknowledged that this was complex and haphazard. They described selectively and consciously paying attention, using retention strategies, reproducing observed behaviour and being motivated to imitate.	-	13

		students and five clinical teachers were purposefully sampled and interviewed. Interviews were audio recorded and transcribed. The data were then analysed using open and axial coding before codes were combined to develop broader themes.	Students evidenced the powerful impact of direct and vicarious reinforcement. Clinical teachers reported using strategies to help students learn, but these were not always consciously or consistently applied or informed by teachers' understanding of their students' cognitive processing.		
Jagsi R. 2014	Quantitative	We obtained Association of American Medical Colleges (AAMC) data about the specialization of 2006-2008 graduates of US medical schools, the sex of their faculty and department chairs, and sex of residents in the residency programs in which they enrolled. We used logistic regression to examine associations between faculty and leadership sex and female students' pursuit of 5 surgical specialties along with 3 nonsurgical specialties for context. We used Wilcoxon rank-sum tests to evaluate whether women entered residency programs with a higher proportion of female residents.	In 2006e2008, US medical school graduates included 23,642 women. Women were substantially under-represented among residents in neurosurgery, orthopaedics, urology, otolaryngology, general surgery, and radiology; women constituted 47.4% of US graduates specializing in internal medicine and 74.9% in pediatrics. We found no significant associa- tions between exposure to a female department chair and selection of that specialty and no consistent associations with the proportion of female full-time faculty. Compared with male students, female students entered residency programs in their chosen specialty that had significantly higher proportions of women residents in the year before their graduation.	9	-
Jayasuriya-Illesinghe, V. 2016	Qualitative	Using qualitative methods we conducted an exploratory study. Twenty eight graduates from 3 medical schools participated in individual interviews. Interview recordings were transcribed verbatim and analyzed using qualitative content analysis method.	Emergent themes reveled 2 types of teaching-learning experiences, role modeling, and purposive teaching. In role modelling, students were expected to observe teachers while they conduct their clinical work, however, this method failed to create positive learning experiences. The clinical teachers who predominantly used this method appeared to be 'figurative' role models and were not perceived as modelling professional behaviors. In contrast, purposeful teaching allowed dedicated time for teacher-student interactions and teachers who created these learning experiences were more likely to be seen as 'true' role models. Students' responses and reciprocations to these interactions were influenced by their perception of teachers' behaviors, attitudes, and the type of teaching-learning situations created for them.	-	15

Appendix A. Tabulated Summaries

Jochemsen-van der Leeuw H. G. A. R. 2013	Systematic review	The authors searched the MEDLINE, EMBASE, ERIC, and PsycINFO databases from their earliest dates until May 2011. They included quantitative and qualitative original studies, published in any language, on role modeling by clinical trainers for trainees in graduate medical education. They assessed the methodological quality of and extracted data from the included studies, using predefined forms.	Seventeen articles met inclusion criteria. The authors divided attributes of role models into three categories: patient care qualities, teaching qualities, and personal qualities. Positive role models were frequently described as excellent clinicians who were invested in the doctor–patient relationship. They inspired and taught trainees while carrying out other tasks, were patient, and had integrity. These findings confirm the implicit nature of role modeling. Positive role models' appearance and scientific achievements were among their least important attributes. Negative role models were described as uncaring toward patients, unsupportive of trainees, cynical, and impatient.	-	-
Jochemsen-van der Leeuw H. G. A. R. 2015	Quantitative	First-year general practitioner (GP) trainees at two institutes for GP speciality training in the Netherlands were asked to complete an assessment of their clinical trainers: the Role Model Apperception Tool (RoMAT). The RoMAT consists of attributes of positive role modelling divided into two components (Caring Attitude and Effectiveness) and was scored on a 5-point Likert scale twice. After the first assessment moment, the trainers received their personal scores combined with the mean score of their peers. The trainers were divided into three performance groups: below average, average and above average.	Only the group with the lowest scores showed an improvement on the Effectiveness component of the Ro-MAT from 3.89 to 4.08 ($p = 0.04$) with an effect size of.52, showing a large effect. This pattern is confirmed by the number of trainers shifting from the below average performance group to the average (7) and above average (5) performance groups.	9.5	-
Keis O. 2019	Quantitative	We recruited 96 students (mean age: 23.83 years; 75% female) in their 5th to 9th semesters at the Faculty of Medicine at the University of Ulm, Germany, who were participating in a clinical placement between July and October 2015. Participants completed a questionnaire at the beginning of a 5-day working week to record sociodemographic and other information and another one at the end of the week to assess various aspects of their experiences. On each of the	Role models and role modelling play an important role in clinical placements. The positive function of medical staff as role models predominated (88.4%) across all specialties. Junior doctors were the most frequently perceived role models (28.5%), followed by consultants (25.1%) and nursing staff (22.4%). The most commonly perceived positive quality was the interaction with students (16.5%), followed by team behaviour (13.6%), interaction with patients (13.6%) and professional	7.5	-

Appendix A. Tabulated Summaries

		5 days, they completed a structured questionnaire to record their perceived role models and self-assessed learning gains.	expertise (13.4%). Students also had various kinds of learning gains such as knowledge or skills.		
Koh G. C. H. 2015	Quantitative	Final year medical students of the Yong Loo Lin School of Medicine, National University of Singapore, from the classes of 2005 (pre- and post-housemanship) and class of 2009 (pre-housemanship) responded to an anonymous 25-statement questionnaire reflecting Fones et al's 25-item characterisation of a "role model" doctor. Qualitative data was also collected on student's perceived qualities of a role model doctor.	For the 2005 cohort pre- and post-housemanship, only 3 of the 25 items had increased in importance post-housemanship. However, when comparing the 2005 and 2009 cohorts pre-housemanship, the latter cohort placed significantly greater importance on 12 of the 25 items. Willingness to teach was identified via qualitative analysis as a new important quality of a role model doctor for medical students.	11	-
Kravet S. J. 2011	Quantitative	Two independent surveys were administered to clinically active faculty (asked to name clinically excellent colleagues) and internal medicine residents (asked to name faculty role models). We compared frequency counts of clinically excellent faculty mentioned and frequency counts of role models mentioned by respondents. Spearman correlations and odds ratios with 95% confidence intervals were used to assess the relationship between the responses.	A total of 39 of 66 faculty (59%) and 45 of 50 residents (90%) responded. There were 31 faculty members judged to be clinically excellent and 67 faculty identified as role models. Thirty faculty members appeared on both lists. There was a moderately high correlation between these groups (Spearman correlation coefficient 5 0.54, P ,.001). Faculty members who were among those named as clinically excellent by their peers were more likely to be named 3 or more times as a role model by trainees (odds ratio, 24.6; confidence interval, 2.9–207).	7.5	-
Kutob R. M. 2006	Quantitative	A questionnaire on medical school experiences and attitudes was administered to primary care graduates from 24 US medical schools.	Questionnaires were completed by 1,457 physicians. Sixty-three percent of primary care respondents had a role model. Having a role model was significantly related to current specialty and ethnicity. Respondents most valued their role models' patient relationships. For family medicine and internal medicine graduates, having a role model was related to more contact and more-positive views of faculty in their specialty. Those with a role model reported that primary care was encouraged at their medical school and were more satisfied with their specialty choice.	8.5	-
Leep A. N. 2017	Quantitative	Students at 10 U.S. medical schools were surveyed in 2015. Thirty-five items assessed attitudes toward, perceived barriers to and consequences of, and	Of 5,992 students invited, 3,395 (57%) responded. Ninety percent (2,640/2,932) agreed physicians have a responsibility to contain costs. However, 48%	8.5	-

		observed physician role-modeling behaviors related to cost-conscious care (using scales for cost-conscious and potentially wasteful behaviors; Cronbach alphas of 0.82 and 0.81, respectively). Regional health care intensity was measured using Dartmouth Atlas Endof- Life Chronic Illness Care data: ratio of physician visits per decedent compared with the U.S. average, ratio of specialty to primary care physician visits per decedent, and hospital care intensity index.	(1,1416/2,960) thought ordering a test is easier than explaining why it is unnecessary, and 58% (1,685/2,928) agreed ordering fewer tests will increase the risk of malpractice litigation. In adjusted linear regression analyses, students in higher health- care-intensity regions reported observing significantly fewer cost-conscious role-modeling behaviors: For each one-unit increase in the three health care intensity measures, scores on the 21-point costconscious role-modeling scale decreased by 4.4 (SE 0.7), 3.2 (0.6), and 3.9 (0.6) points, respectively (all P < .001).		
Lehmann L. S. 2018	Position paper	-	-	-	-
Leman M. A. 2020	Qualitative	We used a grounded theory approach with in-depth interviews and e-mail communications to 48 medical teachers from various backgrounds of "health professions education," "health education and behavior"/'health education and promoter,' "general practitioners/family medicine," "adolescent health," "internal medicine," and "cardiologyvascular medicine." The medical teachers were from Indonesia, one other developing country (Bangladesh), and five developed countries (United States of America, Canada, Netherlands, Australia, and United Kingdom). We also invited 19 medical students from Indonesia for three focus group discussions.	We identified four categories to define a "healthy role-model" for medical schools as persons who are seen: 1) "physically," "socially," "mentally", and "spiritually" healthy; 2) internalized healthy behaviors; 3) willing to promote healthy lifestyles; and, 4) a life-long learner. In each category, there are several characteristics discussed.	-	17
Leman M. A. 2021	Qualitative	This qualitative study involved semi-structured in-depth interviews with medical teachers categorized as healthy role models in a medical school from a previous survey. Ten medical teachers were selected using purposive sampling. Three medical teachers were interviewed by direct meetings, and the remaining were phone interviewed, with one interview facilitated by chat using WhatsApp. Tran-scribed interviews were coded openly. Themes	Two themes were identified: perceived facilitators and perceived barriers, which were classified into four cate-gories and 13 subcategories: intrinsic facilitators (motivation, conscious awareness, having physical limitations, knowledge, and economic reasons); extrinsic facilitators (the impact on doing a particular job, feedback, time, and envi-ronment); intrinsic barriers (the lack of self-motivation and having physical limitations);	-	19

Appendix A. Tabulated Summaries

		were finalized through discussion and debate to reach a consensus.	and extrinsic barriers (the bur-den of responsibilities for being medical teachers and envi-ronment).		
Levine M. P. 2015	Opinion	-	-	-	-
Lindberg O. 2020	Qualitative	This study employed an explorative, qualitative, and cross-sectional design. A total of 57 interviews were held with medical students (28 interviews) and with faculty mem-bers (29 interviews) at a Swedish medical school. Participants were asked to describe their role models and the attributes that made certain individuals role models. Data were ana-lysed using an inductive approach in three separate steps that explored the relationship between role models and gender.	Males do not generally consider female doctors as role models, and male role models are generally viewed as more admirable than female role models. This was shown in all steps of the analysis and most prominently in how male role models were described as qualitatively more admirable than female role models. Male role models are thus more common (for male and female students) and described as more admirable. The results point to the persistence of 'gen-dered ways of thinking' that subtly shape medical students.	-	12
Maker V. K. 2004	Quantitative	Fourth- and fifth-year surgical residents from 1 residency program were asked to collaboratively define 9 characteristics that make a surgical role model. The 9 criteria as defined by the residents were didactic teaching, teaching rounds, attendance at didactic activities, demonstrates skills and decision making in the operating room with confidence and virtuosity, allows [resident] to do procedures according to ability, allows autonomy to make independent decisions, provides feedback, stimulates critical thinking with use of literature, and assists [resident] to find and complete research for publication. Each resident in the program was then given a questionnaire and asked to evaluate each of the 49 teaching attendings on each of the 9 criteria, on a 3-point scale. Finally, residents evaluated each attending based on professionalism and mutual respect on a scale of I Don't Want To Emulate Him/Her, OK, or Role Model. These categories were also assigned a number (1, 2, and 3, respectively). Pearson correlation and stepwise multiple regression were used to determine the	A total of 847 questionnaires were analyzed. Each of the 9 criteria correlated significantly with the Role Model rating (all p 0.01). The average correlation was 0.73 (range, 0.64 to 0.78). Of the 9 criteria, 4 correlated best with the Role Model, as shown in Table 1. The stepwise regression indicates that 3 of the 9 criteria are uniquely associated with the Role Model variable. These 3 criteria are stimulates critical thinking with use of literature, allows autonomy to make independent decisions, and attendance at didactic activities.	7	-

Appendix A. Tabulated Summaries

		relationship between “Role Model” and the 9 criteria. The unit of analysis was the mean rating given each attending on each of the 10 scales.			
Malpas P. J. 2012	Opinion	-	-	-	-
Marisette S. 2020	Qualitative	This study employed a qualitative description design. The CanMEDS– Family Medicine 2009 framework was used to help design interview questions. Interviews were audiorecorded and transcribed verbatim. Transcripts were coded and themes were developed.	Some residents described insufficient experience with role modeling in general. Two main findings were that a longitudinal relationship with a role model was important and that residents desired a close working relationship with a role model in a clinical setting. Most participants could identify experiences with role modeling of ethical practice; many examples were in the context of challenging patients. Some, but not all, residents could identify experiences with role modeling of profession-led regulation and reflective practice. Of note, there were mixed responses with respect to role modeling a commitment to personal health.		16
Martinez W. 2014	Quantitative	Between May 2011 and June 2012, 435 residents at two large academic medical centers and 1,187 medical students from seven U.S. medical schools received anonymous, electronic questionnaires. The questionnaire asked respondents about (1) experiences with errors, (2) training for responding to errors, (3) behaviors related to error disclosure, (4) exposure to role-modeling for responding to errors, and (5) attitudes regarding disclosure. Using multivariate regression, the authors analyzed whether frequency of exposure to negative and positive role-modeling independently predicted two primary outcomes: (1) attitudes regarding disclosure and (2) nontransparent behavior in response to a harmful error.	The response rate was 55% (884/1,622). Training on how to respond to errors had the largest independent, positive effect on attitudes (standardized effect estimate, 0.32, P < .001); negative rolemodeling had the largest independent, negative effect (standardized effect estimate, −0.26, P < .001). Positive role-modeling had a positive effect on attitudes (standardized effect estimate, 0.26, P < .001). Exposure to negative role-modeling was independently associated with an increased likelihood of trainees’ nontransparent behavior in response to an error (OR 1.37, 95% CI 1.15–1.64; P < .001).	10	-
Maudsley R. F. 2001	Commentary	-	-	-	-
McLean M. 2006	Quantitative	Information on role models was collected in the same manner as previously described, by means of	More PBL students (73.6% average) had role models than did students in either of the first two years of the	7	-

Appendix A. Tabulated Summaries

		an anonymous questionnaire (containing open- and closed-ended statements) administered at the end of the academic year (McLean, 2004a). In order to make meaningful comparisons between the junior students in the two curricula, data from students in Years 1 and 2 of the traditional curriculum (2000) were compared with those collected for the first two Year 1 cohorts (2001 and 2002) of the PBL programme.	traditional programme Almost four times as many C2001 students identified faculty role models (including a few research associates) than did their traditional curriculum counterparts (16.5% of PBL vs. 4.6% of traditional curriculum; p< 0.01). There appeared to be no gender differences, as equal numbers of male and female role models were chosen by students in the two curricula. PBL students identified mainly medically qualified individuals (78.8% and 83.6% of the staff identified by the two C2001 cohorts, including the paramedic; 2 and Fisher's exact p < 0.05; Table 3). The few staff members identified by second-year traditional students were invariably their medical science lecturers. Surprisingly, two of the three faculty role models identified by the first-year traditional curriculum students were clinical teachers with whom they had had no contact. More medically qualified role models external to the faculty, although not significant, were identified by C2001 students (8.9% vs. 6.6% for traditional curriculum students). The same trend was noted for 'self ' as a role model and the recognition of different qualities in different people. A significant decline in the family members identified (30.7% of PBL vs. 45.3% of traditional curriculum		
Mileder L. P. 2014	Commentary	-	-	-	-
Mohammadi E. 2020 (a)	Quantitative	Clinical educators were divided into intervention and control groups. The longitudinal program, developed based on the exposure phase of the 'Positive Doctor Role Modelling' framework, was delivered during three months of onsite and online sessions. The effectiveness of the program was assessed in three levels of reaction, learning, and behavior.	In the intervention group (N.18), the mean score of satisfaction was 4.7 (SD.0.5), and the learning (awareness about role modelling) improved significantly after the program (3.33–4.34), comparing to the control group (3.53–3.63). There was no significant difference in terms of behavior improvement between the two groups, before and after the program.	13.5	-
Mohammadi E. 2021	Qualitative	This qualitative study was conducted on eighteen clinical educators who were voluntarily participated in a three-month role modeling educational	Data analysis resulted in the development of three main categories, namely closer attention to role modeling and effort for its promotion, deliberate effort to display role	-	8

Appendix A. Tabulated Summaries

		program. Data were collected using reflection paper writing and were analyzed through conventional content analysis.	modeling, and creating a positive environment to increase the effectiveness of role modeling.		
Mohammadi E. 2020 (b)	Systematic review	We performed a review search using specific keywords (curriculum, role model*, faculty development, teach*, program* and education) through electronic databases (PubMed, EMBASE, and ERIC). We obtained 320 qualitative and quantitative studies. Having removed the duplicate references, we read 244 titles and excluded irrelevant ones. Eighty-two articles were retained and the abstract of each was read. Finally, 20 articles were included.	According to the results of our review, three major themes were identified: 1) features of a good role model composed of teaching, clinical, and personal-interpersonal skills, 2) self-improvement of role modeling, and 3) faculty development programs	-	-
Parmley W. W. 2001	Perspective	-	-	-	-
Passi V. 2016	Qualitative	This study was part of a larger study investigating the process of positive doctor role modeling in medical education. This study used focus group interviews with 52 medical students, semi-structured interviews with 25 consultants and interviews after clinics with five consultants and five medical students. A qualitative methodology using the grounded theory approach of Strauss and Corbin was then used to explore the impact of modeling in medical education.	Three main outcomes of role modeling were identified – the development of professional behaviors, the development of professional identity, and the shaping of career aspirations.		16
Patel M. S. 2015	Quantitative	Cost-conscious care surveys were administered to internal medicine residents during the 2012 Internal Medicine In-Training Examination and to program directors during the 2012 Association of Program Directors in Internal Medicine Annual Survey. Respondents stated whether or not they agreed that faculty in their program consistently role-model cost-conscious care. To evaluate a more comprehensive assessment of faculty behaviors, resident responses were matched with those of the director of their residency program. A multivariate	Among all responses in the final sample, 6,816 (54.0 %) residents and 121 (47.8 %) program directors reported that faculty in their program consistently role-model cost-conscious care. Among paired responses of residents and their program director, the proportion that both reported that faculty do consistently role modeled cost-conscious care was 23.0 % for programs with a formal residency curriculum in cost-conscious care, 26.3 % for programs working on a curriculum, and 23.7 % for programs without a curriculum. In the adjusted model, the presence of a formal curriculum in cost conscious care did not have	7	-

Appendix A. Tabulated Summaries

		logistic regression model was fit to the outcome variable, to identify predictors of responses that faculty do consistently role model cost-conscious care from residency program, resident, and program director characteristics. The primary outcome measure was responses to questionnaires on faculty role-modeling cost-conscious care.	a significant impact on survey responses (odds ratio [OR], 1.04; 95%Confidence Interval [CI], 0.52–2.06; p value [p]=0.91).		
Phelan S. M. 2015	Quantitative	Prospective cohort study of medical students enrolled at 49 US medical schools randomly selected from all US medical schools within strata of public/private schools and region. 1,795 medical students surveyed at the beginning of their 1st year and end of their 4th year. Web-based surveys included measures of weight bias, and medical school experiences and climate. We compared bias change to changes in the general public over the same time period. We used linear mixed models to assess the impact of curriculum, contact with people who have obesity, and faculty role-modeling on weight bias change	Increased implicit and explicit biases were associated with less positive contact with patients who have obesity and more exposure to faculty role-modeling of discriminatory behavior or negative comments about patients with obesity. Increased implicit bias was associated with training in how to deal with difficult patients. On average, implicit weight bias decreased and explicit bias increased during medical school, over a period of time where implicit weight bias in the general public increased and explicit bias remained stable.	10.5	-
Piccinato C. E. 2017	Quantitative + qualitative	Residents from all years of various surgical subspecialties in a university hospital were included in a survey about the factors that determined their choice of surgery. The questions included items on whether a role model had influenced them in choosing surgery, and the personal or professional characteristics of the models that had been most influential. The responses were subjected to qualitative content analysis.	Sixty-four out of 96 medical residents participated. Fifty-three residents (82.8%) acknowledged the influence of role models. Sixteen model characteristics were indicated as important, with 136 mentions. Characteristics classified as technical skills (55%), such as “medical knowledge” and “manual dexterity” predominated over humanistic characteristics (35%), such as “patient-physician relationships” and “ethical behavior”. However, this difference was not statistically significant (Fisher test, P = 0.11). There were no age differences regarding the proportions mentioning “technical” and “non-technical” attributes, but female residents mentioned significantly more technical skills than their male colleagues did.	7.5	5
Pinard A. M. 2018	Perspective	-	-	-	-
Potisek N. M.	Commentary	-	-	-	-

Appendix A. Tabulated Summaries

2019					
Siegel B. S. 2004	Commentary	-	-	-	-
Smith M. D. 2012	Letter	-	-	-	-
Stahn B. 2014	Qualitative	We conducted 14 semi-structured interviews with residents Eppendorf, III. Department of and attending physicians from the departments of Internal Medicine Internal Medicine, Hamburg, (high patient contact) and Laboratory Medicine (low patient contact) at Germany the University Hospital Hamburg-Eppendorf, Germany. We used template analysis to code the interview transcripts and iteratively reduced and displayed the data. Initial codes and concepts were shaped into categories until agreement on the final template was reached.	We identified five main categories of factors that influenced postgraduate specialty selection. Role models with a civilized code of behavior and expertise in their specialty had had the greatest influence on participants' choice of a specialty across generations. Electives and a doctoral thesis project had also influenced participants' decisions, mainly because of meeting a role model in their supervisor. Patient contact and intellectual challenges were identified as contributing factors in the selection of a specialty with high patient contact. As reasons for selecting a university hospital for postgraduate education four categories were identified: the possibility to participate in scientific research, a broad spectrum of activities, personal contacts and future career opportunities.	-	12
Steele M. M. 2013	Quantitative + qualitative	Mixed sampling methodologies including questionnaires (n.175), focus groups (female, n.4; male, n.4), and individual interviews (female n.10; male, n.9) of junior faculty were conducted in clinical departments at one academic health sciences center.	Questionnaire results indicated that having role models increased commitment to an academic career; mentorship experience during residency training was a high incentive to pursue an academic career; and junior faculty did have identifiable mentorship experiences. Focus group results revealed that mentoring as well as the presence of role models a few years ahead of the junior faculty would promote career development. Females preferred similar age role models who spoke the same language, particularly in the area of promotion. Females identified several challenges and issues including a lack of researcher role models, a range of perceptions regarding the merits of formal versus informal mentoring, and the idea that mentors should provide advice on promotion and grants. Males valued advice on finances while females wanted advice on work–life balance.	7.5	12

Appendix A. Tabulated Summaries

Stephens E. H. 2021	Commentary	-	-	-	-
Tagawa M. 2016	Quantitative	Sixth-year medical students were asked to complete questionnaires in 2013 and 2014 regarding encounters with positive or negative RMs, in terms of patient relationships, clinical expertise, teaching ability, and other factors, during clinical training and other situations. Associations between gender, age, admission status, and recognition of self-achievement and joy of learning in relation to RM encounters were then analyzed.	Among 115 students (75 males, 40 females) who completed the questionnaires, 113 (98.3 %) and 85 (73.9 %) reported encountering positive and negative RMs, respectively. The majority of students reported encountering both positive and negative RMs in terms of relationships with patients, humanity, and teaching ability, and fewer negative RMs in terms of clinical expertise and contributions to the community. Older students, males, and those who had passed an entrance examination for bachelors reported encountering more negative RMs in terms of relationships with patients, humanity, and teaching ability than younger students, females, and general admission students. These results suggested an association between positive and negative RM encounters and recognition of self-achievement and joy of learning in formal clinical training.	7.5	-
Tariq S. G. 2016	Qualitative	Seven focus groups were conducted with 82 medical students after they completed the LEP survey. Analysis of focus group transcripts was performed to better understand the nature and meaning that students ascribe to derogatory comments.	The study results provide insights into the types of derogatory comments that medical students heard during their clerkship rotations, why the comments were made and how they were interpreted. Emergent themes, labeled by the authors as 1) 'onstage-offstage', 2) 'one bad apple', and 3) 'pressure cooker environment', highlight the contextual aspects and understandings ascribed by students to the derogatory comments. Incidentally, students felt that the comments were not associated with fatigue, but were associated with cumulative stress and burn-out.	-	10
Teng V. C. 2014	Editorial	-	-	-	-
Wittlin N. M. 2019	Quantitative	Data were collected by surveying students (n=2940) from a stratified sample of U.S. medical schools in fall 2010 (first semester of medical school), spring 2014 (final semester of medical school), and spring 2016 (second year of medical residency).	Amount and favorability of contact with LGBT individuals, reported during the final semester of medical school, predicted lower levels of explicit bias against lesbian and gay individuals during second year of medical residency. Additionally, exposure to negative role modeling, also reported during the final semester of medical school,	13	

Appendix A. Tabulated Summaries

			predicted higher levels of explicit bias against lesbian and gay individuals during second year of medical residency. Amount of contact with LGBT individuals – and in particular, with LGBT medical students – predicted lower levels of implicit bias against lesbian and gay individuals during second year of medical residency. Neither favorability of contact with LGBT individuals nor exposure to negative role modeling predicted implicit bias against lesbian and gay individuals during second year of medical residency.		
Wong A. 2014	Qualitative	Sixty-five medical students (46 women; 19 men) from a class of 194 consented to the study of their portfolios. In total, 604 reflections were analysed and coded using thematic narrative analysis. The codes were merged under subthemes and themes. Common or recurrent themes were identified in order to develop a descriptive framework of professional identity formation. Reflections were then analysed longitudinally within and across individual portfolios to examine the professional identity formation over time with respect to these themes.	Five major themes were associated with professional identity formation in medical students: prior experiences, role models, patient encounters, curriculum (formal and hidden) and societal expectations. Our longitudinal analysis shows how these themes interact and shape pivotal moments, as well as the iterative nature of professional identity from the multiple ways in which individuals construct meaning from interactions with their environments.	-	9
Yazigi A. 2016	Letter	-	-	-	-
Yoon J. D. 2018	Quantitative	We conducted a 5-year prospective, national longitudinal study (2011–2016) of medical students from 24 US allopathic medical schools, starting from the middle of their third year. The primary outcome measure was type of residency specialty choice 4 years after graduation. Main predictors were the clinical specialty of a student's most admired physician and the relative importance of 7 potentially influential factors for specialty choice in the fourth year of medical school.	From 919 eligible participants, 564 (61%) responded to the first survey; 474 of the respondents (84%) completed the follow-up survey. We excluded 29 participants who were not in their fourth year by the time of the follow-up survey. Of the followup respondents, 427 (96%) had specialty data 4 years after graduation. In our multivariate models, exposure to an admired generalist physician prior to medical school (odds ratio [OR] . 2.21, 95% confidence interval [CI] 1.03–4.73) and during medical school (OR . 2.62, 95% CI 1.69–4.05) had the strongest odds with respect to training in a generalist residency 4 years after	11	-

			graduation. Role model exposure also predicted specialty choice among those training in surgical and radiology, ophthalmology, anesthesiology, and dermatology (ROAD) specialties.		
